# Do COVID-19 CT features vary between patients from within and outside mainland China? Findings from a meta-analysis

**DOI:** 10.3389/fpubh.2022.939095

**Published:** 2022-10-14

**Authors:** Nianzong Hou, Lin Wang, Mingzhe Li, Bing Xie, Lu He, Mingyu Guo, Shuo Liu, Meiyu Wang, Rumin Zhang, Kai Wang

**Affiliations:** ^1^Center of Gallbladder Disease, Shanghai East Hospital, Institute of Gallstone Disease, School of Medicine, Tongji University, Shanghai, China; ^2^Department of Hand and Foot Surgery, Zibo Central Hospital, Shandong First Medical University and Shandong Academy of Medical Sciences, Zibo, China; ^3^Department of Critical Care Medicine, Zibo Central Hospital, Shandong First Medical University and Shandong Academy of Medical Sciences, Zibo, China; ^4^School of Molecular and Cellular Biology, University of Leeds, Leeds, United Kingdom; ^5^Department of Urology, Dongfeng Hospital, Hubei University of Medicine, Shiyan, China; ^6^Department of Cardiology, The People's Hospital of Zhangdian District, Zibo, China

**Keywords:** chest CT, COVID-19, diagnosis, meta-analysis, SARS-CoV-2

## Abstract

**Background:**

Chest computerized tomography (CT) plays an important role in detecting patients with suspected coronavirus disease 2019 (COVID-19), however, there are no systematic summaries on whether the chest CT findings of patients within mainland China are applicable to those found in patients outside.

**Methods:**

Relevant studies were retrieved comprehensively by searching PubMed, Embase, and Cochrane Library databases before 15 April 2022. Quality assessment of diagnostic accuracy studies (QUADAS) was used to evaluate the quality of the included studies, which were divided into two groups according to whether they were in mainland China or outside. Data on diagnostic performance, unilateral or bilateral lung involvement, and typical chest CT imaging appearances were extracted, and then, meta-analyses were performed with R software to compare the CT features of COVID-19 pneumonia between patients from within and outside mainland China.

**Results:**

Of the 8,258 studies screened, 19 studies with 3,400 patients in mainland China and 14 studies with 554 outside mainland China were included. Overall, the risk of quality assessment and publication bias was low. The diagnostic value of chest CT is similar between patients from within and outside mainland China (93, 91%). The pooled incidence of unilateral lung involvement (15, 7%), the crazy-paving sign (31, 21%), mixed ground-glass opacities (GGO) and consolidations (51, 35%), air bronchogram (44, 25%), vascular engorgement (59, 33%), bronchial wall thickening (19, 12%), and septal thickening (39, 26%) in patients from mainland China were significantly higher than those from outside; however, the incidence rates of bilateral lung involvement (75, 84%), GGO (78, 87%), consolidations (45, 58%), nodules (12, 17%), and pleural effusion (9, 15%) were significantly lower.

**Conclusion:**

Considering that the chest CT features of patients in mainland China may not reflect those of the patients abroad, radiologists and clinicians should be familiar with various CT presentations suggestive of COVID-19 in different regions.

## Introduction

The epidemic of coronavirus disease 2019 (COVID-19), originally occurred in Wuhan, Hubei, China, caused by severe acute respiratory syndrome coronavirus 2 (SARS-CoV-2) has spread worldwide (1, 2). As a public health emergency, COVID-19 is still an ongoing outbreak, and humans have been experiencing a relentless spread of variations of SARS-CoV-2 (3, 4), significantly undermining the domains of health, economy, environment, and society, unfortunately (5).

Given the fact that the transmission ability of COVID-19 is stronger and the incidence of mortality is relatively higher (6, 7), rapid and accurate diagnostic methods show their great significance for the prevention, control, and management of COVID-19. Real-time fluorescence polymerase chain reaction (RT-PCR) remains the current gold standard for COVID-19 diagnosis (8); however, this diagnosis method has some shortcomings: (1) it is very time-consuming to obtain the results after sampling and may lead to experimental errors caused by manual handling (9, 10), (2) there are a high rate of false-negative results (11), where there were even repeated negatives confirmed by other methods for patients when viral load is insufficient (12), (3) not all hospitals and clinics can implement these methods or the supply and quality of the reagents cannot keep up with the demand in time (13, 14), (4) the severity, progression, and the evaluation of patients cannot be judged or traced (11, 15, 16). Compared to several limitations mentioned above, chest computerized tomography (CT), as a routine and powerful tool for diagnosing viral pneumonia, has the advantage of timeliness, celerity, and high stability and sensibility (17, 18). The superiorities of chest CT in the diagnosis of COVID-19 have been approved and can be used as a quick and efficient method to detect COVID-19 (17–20), which is a significant alternative to RT-PCR testing for early diagnosis (17, 19, 21), especially when the results of RT-PCR are postponed or capacities are finite (22) or when the SARS-CoV-2 infection needs to be ruled out rapidly in those patients involved in emergency surgery (23, 24).

Nevertheless, a study by Kim et al. (25) observed that chest CT screening of patients with suspected COVID-19 had a low positive predictive value in a low prevalence region; several reviews and meta-analysis demonstrated that the considerable variation in the prevalence of disease severity and mortality was across different geographic regions (26–28). In a case-control study, Zhang et al. (29) discovered that a few patients with COVID-19, out of Wuhan but, from China lacked typical CT manifestations. All of the studies mentioned above indicated that the chest CT features of patients with COVID-19 may vary across different countries, territories, and regions. Hence, a comprehensive understanding of the suggestive features of chest CT based on specific countries or regions could help us to differentiate COVID-19 pneumonia and screen highly suspicious cases. Furthermore, explicitly stating whether chest CT findings of patients with COVID-19 in mainland China are applicable to those outside mainland China could provide indirect evidence, which contributes to the exchange of opinions and information, defeats the purpose of settling disputes, and adds to the literature on the CT performance of patients with SARS-CoV-2 infection, potentially enhancing the understanding of COVID-19. To the best of our knowledge, the distinctiveness of chest CT features has not been observed between patients with COVID-19 from within and outside mainland China.

We performed this meta-analysis, with the primary objective of quantitatively summarizing the results from published studies to date to compare and assess the differences in the diagnostic value and appearances of chest CT between patients with COVID-19 from within and outside mainland China, to provide a more precise estimate in detecting patients with COVID-19 in different regions. Our secondary objective of the systematic review was an attempt to clarify what causes the differences in chest CT appearances between patients with COVID-19 from within and those outside mainland China.

## Methods

### Search strategy and study selection

We searched PubMed, Embase, and Cochrane Library for studies reporting chest CT features of patients with COVID-19 published online before 15 April 2022. Search terms included (2019-nCoV) OR (2019 Novel Coronavirus) OR (COVID-19) OR (SARS-CoV-2) AND (chest CT), which were used as the subject or free words adjusted according to the different characteristics of the databases involved. We also manually searched the references of the studies included to retrieve any eligible studies. In addition, only articles in English were included. Two authors (LW and ML) independently screened the titles and abstracts and then carefully read the full texts to select suitable articles according to the inclusion and exclusion criteria. These included articles were separated into two groups according to patients in or outside mainland China. Meta-analyses of the two groups were performed using the Preferred Reporting Items for Systematic Reviews and Meta-Analyses (PRISMA) guidelines (30).

### Inclusion and exclusion criteria

The following inclusion criteria were used to identify all eligible studies: (1) full-text original articles in English, (2) the study population including patients diagnosed with COVID-19, (3) cohort studies, case-control studies, or case studies consisting of at least five patients, (4) at least one of the observational indicators having chest CT features of COVID-19, and (5) the number of corresponding imaging features extractable in this study.

The exclusion criteria were (1) duplicate studies or study populations completely overlapping other studies, (2) full-texts nonaccessible or with a sample size <5, (3) studies that only reported the specificity or sensibility of chest CT, (4) patients that could not be labeled within or outside mainland China, (5) studies on pregnant women, and (6) corresponding outcome parameters or necessary data could not be acquired or separated even by contacting the author.

### Data extraction and quality assessment

Two researchers independently extracted the following information from each included article: the first author's full name, countries/regions of patients, study sample size, diagnostic criteria, experimental design method, mean or median age, gender, the application of special CT or not, CT imaging manifestations, and the number of patients with abnormal CT results. If there was a disagreement, it was resolved by discussion or consultation with the third author. The content of the recorded lesion patterns on chest CT mainly included the following aspects: ground glass opacities (GGO), consolidations, GGO mixed consolidations, the crazy-paving sign, linear opacities, nodules, tree-in-bud appearance, air bronchogram, halo sign, adjacent pleural thickening, septal thickening, lymphadenopathy, pericardial effusion, pleural effusion, and vascular engorgement (bilateral or unilateral lungs). Because the included articles in this research were observational studies, we utilized the quality assessment of diagnostic accuracy studies (QUADAS) scale to evaluate study quality by two independent reviewers (BX and LH) (31).

### Statistical analysis

Corresponding data from the two groups (patients in and outside mainland China) in the meta-analyses were pooled using single-arm analyses. Because some related data extracted from the original articles were too volatile, we used the double arcsine method to transform the incidence rates to a normal distribution, and then the transformed data were used in meta-analyses. We conducted the *I*^2^ statistics to analyze heterogeneity between the studies, and heterogeneity was considered significant or severe if the value of *I*^2^ was >50%, and in this situation, a random-effects model was utilized; otherwise, a fixed effects model was suitable when no statistical heterogeneity was observed. Pooled data for the results of meta-analyses were recalculated using the formula {*p* = [sin(tp/2)]2}. Publication bias was evaluated using Egger's regression test, and a *p*-value < 0.10 was statistically significant. Chest CT characteristics in the two groups were compared using the χ^2^ test or the Fisher's exact test when data were limited. A two-tailed *p*-value < 0.05 was considered statistically significant. All statistical analyses were performed using the *R* statistical computing language, version 3.6.1 (The R Foundation for Statistical Computing, Vienna, Austria, https://www.r-project.org).

## Results

### Study selection and characteristics

**Figure 1:**
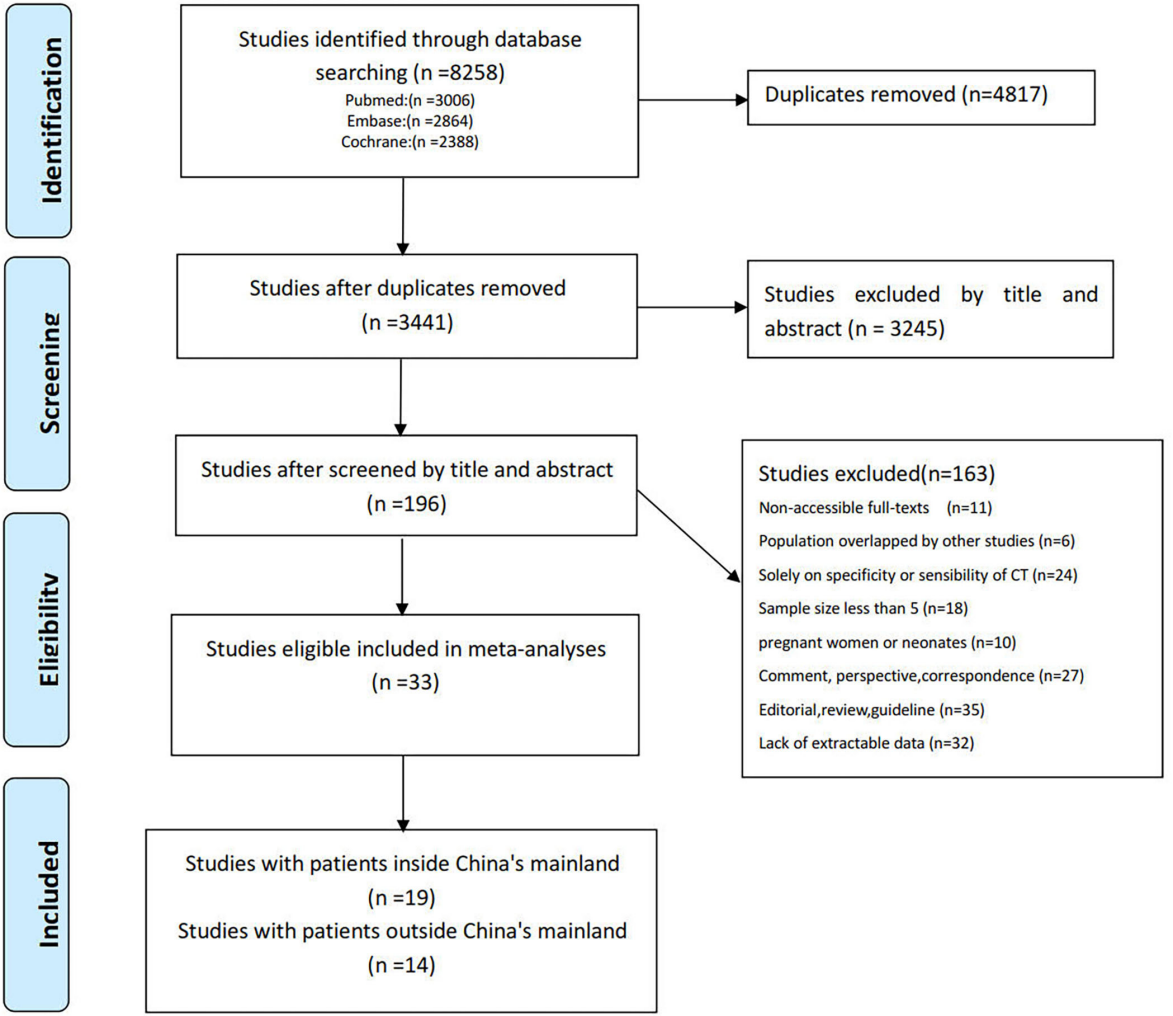
Preferred Reporting Items for Systematic Reviews and Meta-Analyses guidelines (PRISMA) flow diagram of the study selection process.

Totally, 8,258 studies published online before 15 April 2022 were identified along with the search strategy and 3,441 studies remained after the exclusion of duplicates. Then, 3,245 studies were excluded by the title and abstract. After full-text review, 163 studies were excluded for the following reasons: full text not accessible (*n* = 11), population overlapping with other studies (*n* = 6), specificity or sensibility of CT alone (*n* = 24), sample size <5 (*n* = 18), studies on pregnant women (*n* = 10), comment, perspective, and correspondence (*n* = 27), editorial, review, and guideline (*n* = 35), and lack of extractable data (*n* = 32). Consequently, the remaining 33 independent studies that satisfy the inclusion and exclusion criteria were used in the current analyses, which had 3,954 patients with 3,449 having one or more abnormal CT imaging features and nearly all CT scans were carried out within 7 days after the onset of symptoms. Of these studies, 14 studies (32–45) with 554 participants reported at least one abnormal chest CT performance in 453 patients outside mainland China (i.e., Japan, USA, Australia, Tunisia, Turkey, Iran, Mexico, Brazil, Hong Kong, Taiwan, etc.) and 19 studies (16, 17, 46–62) with 3,400 patients reported at least one abnormal chest CT performance in 2,996 patients in mainland China. A detailed search procedure is summarized in Figure 1, and the characteristics of the included studies are outlined in Supplementary Table S1.

### Quality assessment

The analysis of the QUADAS scale showed that the reporting quality of the articles included was better (Supplementary Table S2). Most of the included studies had a relatively low risk of bias in patient selection, index tests, reference standards, and patient flow. Meanwhile, the paucity of details in some studies raised a few concerns regarding applicability in those descriptions (Supplementary Figure S1).

### Meta-analyses results

There was substantial heterogeneity in most analyses of chest CT for patients with COVID-19 in different countries and regions; therefore, the random effects models were used in most of these meta-analyses. However, air bronchogram and nodule analyses in patients outside mainland China used the fixed-effects models in which heterogeneity was less obvious.

### Diagnostic value of chest CT

**Figure 2:**
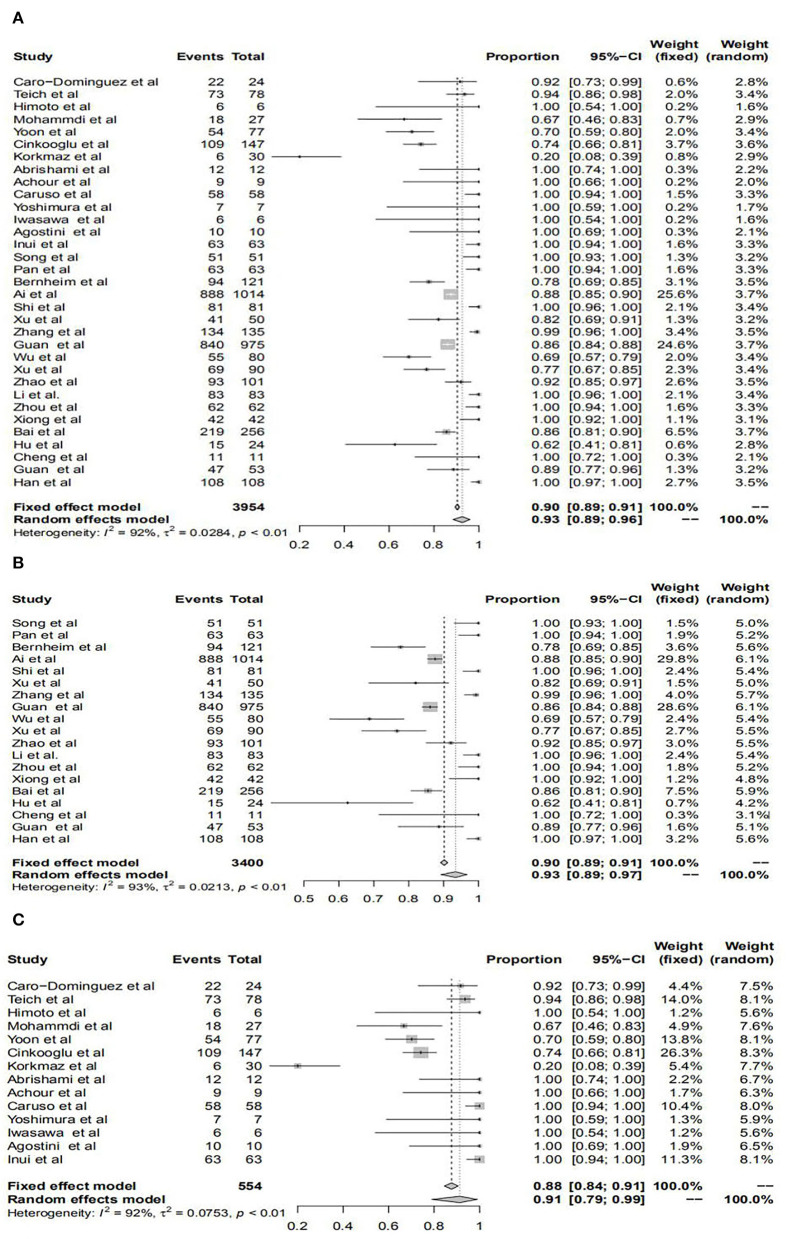
Forest plots of the diagnostic performance of chest computerized tomography (CT) in studies included in the meta-analyses. Forest plots show the prevalence of positive chest CT for all patients **(A)**, patients within mainland China **(B)**, and patients outside mainland China **(C)**.

The pooled prevalence of positive chest CT for all patients was 93%, for patients in mainland China was 93%, and for patients outside was 91%, respectively. However, there was no significant difference in the diagnostic value of chest CT between patients from within and those outside mainland China. All results are shown in Figure 2 and Table 1.

**Table 1:** Computerized tomography (CT) imaging findings in different analyses and comparisons in patients from within and outside mainland China.

	**Overall patients**		**Patients in**		**Patients outside**		**Chi-squared test/**	
			**mainland China**		**Mainland China**		**Fisher exact test**	
	**Pooled**	**95% CI**	**Pooled**	**95% CI**	**Pooled**	**95% CI**	**χ^2^**	***p***
	**Proportion**		**Proportion**		**Proportion**			
Abnormal CT	93%	89–96%	93%	89–97%	91%	79–99%	2.60	0.110
Bilateral lung	78%	69–86%	75%	65–84%	75%	65–84%	13.14	< 0.010
Unilateral lung	12%	7–17%	15%	8–23%	7%	2–13%	17.44	< 0.010
GGO	82%	73–89%	78%	68–88%	87%	70–98%	22.81	< 0.010
Consolidations	50%	43–57%	45%	39–52%	58%	38–76%	27.45	< 0.010
Crazy-paving sign	26%	14–40%	31%	11–55%	21%	8–38%	10.21	< 0.010
Mixed GGO and consolidation	45%	36–54%	51%	41–61%	35%	24–46%;	17.65	< 0.010
Air bronchogram	38%	24–53%	44%	23–65%	25%	20–30%	33.80	< 0.010
Nodules	14%	7–23%	12%	5–23%	17%	12–23%	4.01	0.045
Vascular engorgement	42%	27–58%	59%	45–72%	33%	11–59%	49.91	< 0.010
Bronchial wall thickening	16%	9–25%	19%	9–31%	12%	1–30%	5.60	0.018
Septal thickening	35%	21–50%	39%	20–59%	26%	10–45%	20.04	< 0.010
Pleural effusion	11%	5–18%	9%	5–13%	15%	0–43%	8.11	0.004
Halo sign	22%	8–42%	–	–	–	–	–	–
Linear opacities	45%	31–59%	–	–	–	–	–	–
Lymphadenopathy	14%	2–32%	–	–	–	–	–	–
Pleural thickening	26%	12–42%	–	–	–	–	–	–

GGO, ground-glass opacities; CI, confidence interval.

### Unilateral and bilateral lung infection manifested by chest CT

**Figure 3:**
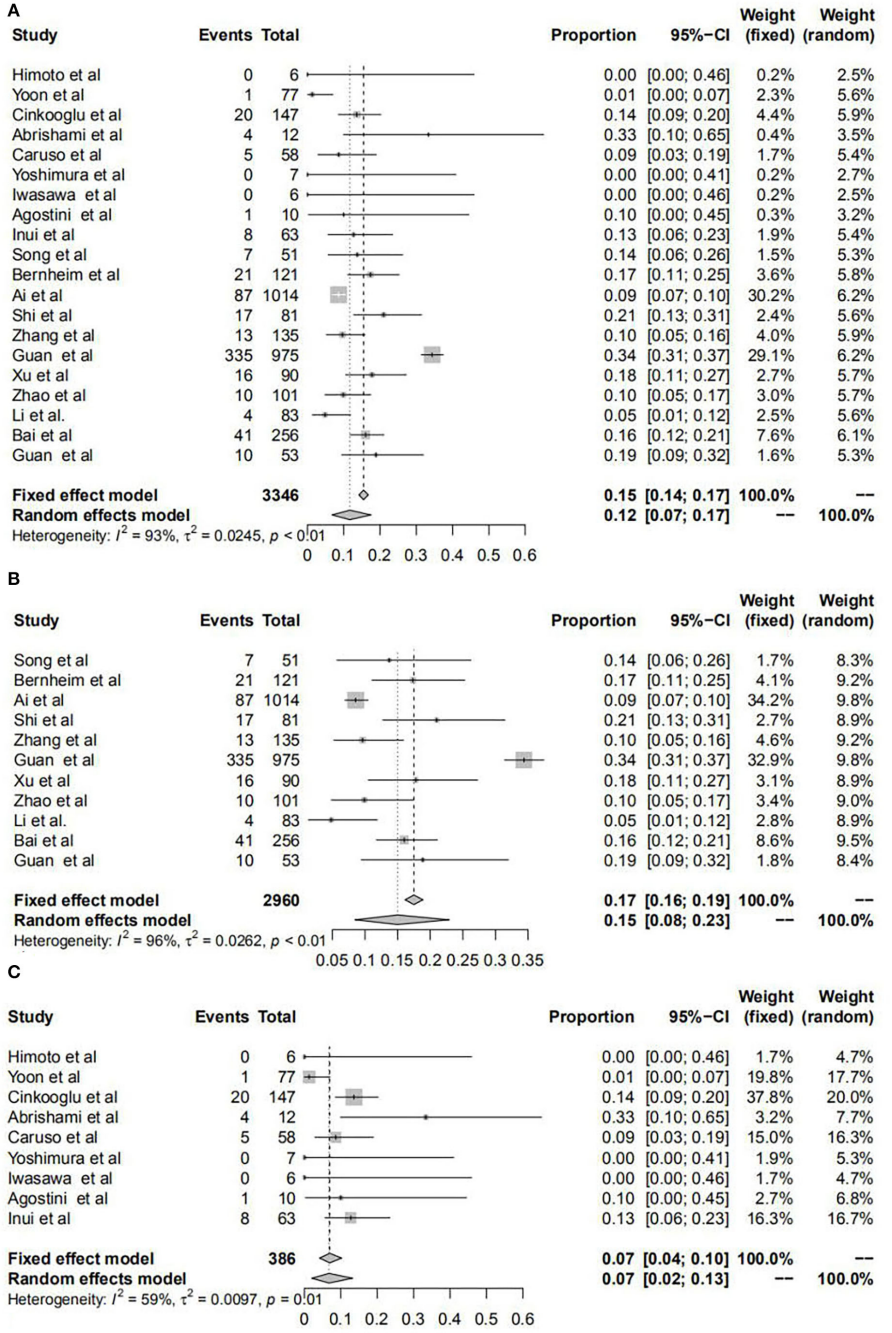
Forest plots of unilateral lung infection manifested by chest CT for patients with COVID-19. Forest plots show the transformed incidence rate of unilateral lung involvement in all patients **(A)**, patients within mainland China **(B)**, and patients outside mainland China **(C)**. COVID-19: coronavirus disease 2019; CI, confidence interval.

The pooled transformed incidence rates of unilateral and bilateral lung involvement in all patients were 12% and 78%, respectively. For patients in mainland China, we found that the incidence rates of unilateral and bilateral lung involvement were 15 and 75%; however, these data were 7 and 84% for the outside, respectively. In addition, through the χ^2^ test, it was proven that there were significant differences in the incidence rates of both unilateral and bilateral lung infection manifested by chest CT for patients in and outside mainland China. All results are shown in Figure 3, Supplementary Figure S2, and Table 1.

### Subgroup analyses of typical CT imaging appearances

We found that typical CT imaging appearances of patients both in and outside mainland China were GGO, consolidations, the crazy-paving sign, mixed GGO and consolidation, air bronchogram, nodules, vascular engorgement, bronchial wall thickening, septal thickening, pleural effusion, halo sign, linear opacities, lymphadenopathy, and pleural thickening. For GGO, the pooled incidence rates were 82% for all patients, 78% for patients in mainland China, and 87% for patients outside mainland China, respectively; for consolidations, the pooled incidence rates were 50, 45, and 58%, respectively; for the crazy-paving sign, the rates were 26, 31, and 21%, respectively; for mixed GGO and consolidation, the rates were 45, 51, and 35%, respectively; for air bronchogram, the rates were 38, 44, and 25%, respectively; for nodules, the rates were 14, 12, and 17%, respectively; for vascular engorgement, the rates were 42, 59, and 33%, respectively; for bronchial wall thickening, the rates were 16, 19, and 12%, respectively; for septal thickening, the rates were 35, 39, and 26%, respectively; for pleural effusion, the rates were 11, 9, and 15%, respectively; and for halo sign, linear opacities, lymphadenopathy, and pleural thickening, the pooled incidence rates of all patients were 22, 45, 14, and 26%, respectively. However, we could not extract the pooled incidence rates for patients in or outside mainland China. All results of subgroup analyses are shown in Supplementary Figures S3–S6 and Table 1.

There were obvious differences in the incidence rates of most typical CT imaging appearances between patients from within and those outside mainland China, and these CT appearances included GGO, consolidation, the crazy-paving sign, mixed GGO and consolidation, air bronchogram, nodules, vascular engorgement, bronchial wall thickening, septal thickening, and pleural effusion. Through the χ^2^ test, the incidence rates of the crazy-paving sign, mixed GGO and consolidation, air- bronchogram, vascular engorgement, bronchial wall thickening, and septal thickening in patients within mainland China were significantly higher than those outside; however, the incidence rates of GGO, consolidations, nodules, and pleural effusion were significantly lower. All results of subgroup analyses are summarized in Table 1.

### Publication bias

The values of *p* derived from Egger's regression asymmetry test for most observational indicators suggested that the publication bias was not obvious (Table 2). There was a low probability of publication bias in the following subanalyses: abnormal CT, bilateral lungs, unilateral lungs, consolidations, the crazy-paving sign, mixed GGO and consolidation, vascular engorgement, bronchial wall thickening, pleural effusion, halo sign, linear opacities, lymphadenopathy, and pleural thickening of overall patients; abnormal CT, bilateral lung, unilateral lung, consolidations, mixed GGO and consolidation, nodules, and vascular engorgement of patients in mainland China; and GGO, consolidations, the crazy-paving sign, mixed GGO and consolidation, air bronchogram, nodules, vascular engorgement, bronchial wall thickening, septal thickening, and pleural effusion of patients outside mainland China. The publication bias of these subanalyses (halo sign, linear opacities, lymphadenopathy, and pleural thickening of patients either in or outside mainland China) could not be evaluated for fewer included studies in each subgroup.

**Table 2:** Egger's test results.

**CT Manifestations**	**Overall patients**		**Patients in**		**Patients outside**	
			**mainland China**		**Mainland China**	
	***T***	***p***	***t***	***p***	***t***	***p***
Abnormal CT	1.11	0.27	1.65	0.12	0.67	0.51
Bilateral lung	1.20	0.25	1.11	0.30	0.43	0.68
Unilateral lung	1.19	0.25	−0.64	0.54	−0.16	0.88
GGO	3.67	< 0.01	3.64	< 0.10	0.34	0.74
Consolidations	1.00	0.32	0.28	0.78	−0.19	0.85
Crazy-paving sign	1.45	0.17	3.64	0.02	1.00	0.36
Mixed GGO and consolidation	0.99	0.36	0.69	0.53	0.43	0.74
Air bronchogram	2.17	0.05	2.28	0.05	1.02	0.38
Nodules	2.22	0.05	1.72	0.12	0.71	0.55
Vascular engorgement	−0.78	0.46	0.70	0.61	−0.57	0.60
Bronchial wall thickening	0.85	0.42	2.46	0.09	−0.03	0.98
Septal thickening	3.44	0.003	3.84	0.004	1.82	0.14
Pleural effusion	1.58	0.13	2.42	0.04	0.40	0.71
Halo sign	−0.40	0.71	–	–	–	–
Linear opacities	−0.06	0.96	–	–	–	–
Lymphadenopathy	1.31	0.25	–	–	–	–
Pleural thickening	0.70	0.52	–	–	–	–

GGO, ground-glass opacities; CI, confidence interval.

## Discussion

In our present study, we focused on investigating the disagreement of typical chest CT characteristics between patients with COVID-19 from within and outside mainland China. Above all, our meta-analysis reinforces the high proportion of COVID-19 detected by chest CT. Our results showed that the pooled incidence rates of abnormal chest CT for all patients were 93% (95% confidence interval (CI): 89–96%), which were similar to several other studies, such as 89.76% (95% CI: 84–94%) by Bao et al. (63) and 94% (95%CI: 91–96%) by Kim et al. (25). This study once again suggested that chest CT could be used as a vital diagnostic tool for COVID-19. Although the pooled incidence rate of abnormal chest CT for patients in mainland China was higher than those outside (93 vs. 91%), this difference did not reach a statistical significance, indicating that chest CT for screening of patients with COVID-19 in different regions deserved recognition.

Regarding bilateral or unilateral lung involvement, we discovered several intriguing results. The pooled incidence rates of bilateral lung involvement in all patients were 78% and indicated that COVID-19 infection most commonly affected bilateral lungs, which was consistent with the results of 78.2% by Bao et al. (63) and 73.8% by Zhu et al. (64). For subgroup analyses, we found that the incidence rates of unilateral lung involvement for patients in mainland China were significantly higher than those outside (15 vs. 7%) and the incidence rates of bilateral lung involvement were significantly low (75 vs. 84%). However, there are no studies addressing these differences in the distribution of COVID-19 infection among patients within different regions. Ooi et al. (65) and Das et al. (66) pointed out that unilateral lung involvement was more common in the early stage of severe acute respiratory syndrome (SARS) and Middle East respiratory syndrome (MERS) and that unilateral involvement of pneumonic infiltrates at a later stage is very rare. However, bilateral lung involvement seemed to be a unique imaging characteristic for COVID-19 although these three respiratory viruses all belong to the family of coronaviruses and the CT features are similar. The results of Bao et al. (63) showed that the incidence of bilateral lung infection had risen to 82% from 78% when they excluded studies without mentioning thin-section chest CT, which might mean that a special CT could show lung changes in more detail. In our meta-analysis, 58% of studies (11/19) for patients in mainland China and 21% (3/14) for those outside reported the use of a special CT for COVID-19 diagnosis, respectively. The higher proportion of the use of special CT in mainland China may help recognize and dismiss the false-positive rate of bilateral lung infection. However, several studies revealed that very basic instruments, low radiation doses, or improper practices might not influence the performance of chest CT in the diagnosis of COVID-19. Dai et al. (67) found that the image quality of the shelter hospital CT (CT Ark) had no obvious difference compared to ordinary CT (Brilliance 64) based on subjective observations. Andrea et al. (68) and Niu et al. (69) recognized that a low radiation dose could still provide sufficient diagnostic quality to exclude COVID-19. Bernardo et al. (70) deemed that there was little difference in the distribution and lobar extent of pulmonary lesions on chest CT despite substantial differences in CT usage. Moreover, the CT images included in the articles were read by two or more senior radiologists with a host of experience. Here, the use of a special CT instrument might not be the cause of such differences.

We also demonstrated that all CT features and corresponding pooled incidence rates were in accordance with previous meta-analyses (63, 64); however, large gaps in the incidence rates of CT features between patients from within and outside mainland China were striking and all differences reached statistical significance. These results that CT patterns of patients with COVID-19 from within mainland China may not reflect those outside mainland China should be interpreted with caution, and the evidence provided to elucidate why features differ between those from within and outside mainland China is indirect and even anecdotal. Because few analyses of anatomical–pathological–radiological correlations have been carried out and studies on the formation of imaging features are scarce and superficial, immune damage from inflammatory responses and deep airway and alveolar epithelial injuries from direct virus attack account for these pathological changes in the lungs (71–73). Angiotensin-converting enzyme-2 (ACE2) is used by SARS-CoV-2 as a cell receptor in human lungs, causing interstitial damage first and parenchymal lesions later (71). We speculate that the coexistence of SARS-CoV-2 with different mutation patterns, race and ethnicity, and other atypical cases of pneumonia may contribute to explain why features differ between patients from within and outside mainland China. In our opinion, pathology is the observation of microanatomy and radiology is the observation of gross anatomy; therefore, CT performances will be different according to the change in viral pathologic manifestations. Wabalo et al. (74) reviewed multiple literature studies and concluded that the high mutation rate of SARS-CoV-2 is likely to change the properties of the virus, including its virulence or infectivity, and may have higher viral loads and develop more severe clinical manifestations. Song et al. (75) found that the infection with the B.1.1.7 variant could lead to a more severe inflammatory response and more severe pneumonia, which implied that this variant might have higher pathogenicity than previous wild type and variants. Zhang et al. (29) found that some of these patients with COVID-19 from other cities in China outside Wuhan lacked the typical CT imaging features, and the results can be attributed to the majority of infections being second-generation human-to-human transmissions. Wu et al. (76) found that the proportion of halo signs and reversed halo signs had increased greatly in patients with SARS-CoV-2 Delta pneumonia. McLaren et al. (77) discovered that the new “bullseye sign,” a variant of the reverse halo sign, could alert clinicians to the possibility of COVID-19, which, we reasoned, might be related to the mutation of the virus. Cheng et al. (78) detected that chest CT changes with the Delta COVID-19 variant were milder than pre-existing strains and considered that the pathological features of the mutated virus could change under the pressure of immune surveillance. Although the radiological severity of alpha variant infection was not increased compared to the original virus, Tsakok et al. (79) showed that the CT severity scores applied to angiography is associated with the COVID-19 outcome. Considering the fact that immune damage is one of the main reasons for pulmonary tissue injury (71–73) and that ethnic differences in immune status in genetic polymorphism are associated with immune-mediated diseases. Therefore, it is reasonable to presume that racial disparity is partly responsible for the different features of chest CT between patients from within and outside mainland China (80). The findings of Ahmed et al. (81) directly showed that there were large variations in radiological manifestations between different ethnic groups, such as Egyptians, Saudis, Indians, Bangladeshis, and so on. In a COVID-19 cohort, Smith et al. (82) observed significant differences in the breadth and strength of the humoral immune response in relation to ethnicity, which might reflect differences in genetic factors. The expression of ACE2, which plays a significant role in determining ethnic susceptibility and protecting pulmonary parenchyma from deterioration due to COVID-19, differs among the world's three main racial groups: Africans, Asians, and Caucasians, revealing that Asians have a significantly higher ACE2 expression in various organs, and the Black population shows a reduced molecular expression of ACE2 compared to other races (83, 84). Furthermore, Li et al. (85) reported that the use of ACE inhibitors/angiotensin receptor blockers (ARBs) was associated with a significant reduction in mortality among African-American patients with COVID-19 positive in the hospital, and Pabalan et al. (86) recommended that ACE2 genotypes might be useful for acute lung injury (ALI)/ARDS therapy for patients with COVID-19. Antoon et al. (87) showed that there were differences in the severity of COVID-19 pneumonia by race and ethnicity but additional research is needed to clarify the sources of such disparities. Meanwhile, Wu et al. found that the 3p21.31 locus is the risk haplotype specific to Europeans and South Asians, but the MEF2B variant specific to East Asians confers an eight-fold increase in the risk of COVID-19 severity (88). In addition, the degree of discrepancies within ethnicity and race also influences the COVID-19 clinical manifestation. For example, Upadhyai et al. (89) revealed that asymptomatic patients with COVID-19 in Europe possessed discernibly higher proportions of the Ancestral North Eurasian, which may be associated with the pathways that govern host immunity, such as interferon, interleukin, cytokine signaling. CT appearances of COVID-19 may overlap or interweave with other viral pneumoniae. Garrana et al. (90–92) reported that the prevalence of influenza viruses, parainfluenza virus, adenovirus, respiratory syncytial virus, rhinovirus, human metapneumovirus, etc. was not the same in different regions, which might be another reason concerning the different features between patients within or outside mainland China. Similar to COVID-19, most cases of viral pneumonia involved both the lungs and multiple lung lobes with a predominant distribution in the posterior and peripheral parts of the lungs; however, CT findings for COVID-19 overlap substantially with those of influenza to a greater extent (93). Li et al. (94) suggested that CT is still limited in identifying specific viruses and distinguishing between the viruses and a new method has been created, which can accurately discriminate COVID-19 from other types of pneumonia (95). Cheng et al. (92) found that patients with COVID-19 with influenza A virus had a significantly lower CT score than those with only SARS-CoV-2 infection, indicating that co-infection may alleviate inflammation in the lungs. Moreover, Zhang et al. (96) emphasized that the pulmonary changes in radiological findings did not show any difference in their location or distribution between patients with COVID-19 and those with seasonal influenza. Investigation of SARS-CoV-2 co-infection with other respiratory viruses is essential to provide novel insights into the development of highly sensitive diagnostics in different regions.

Although the CT findings in patients with COVID-19 are different on various days, it is reasonable to assume that chest CT performance within 7 days is relatively constant and can be compared between patients from within and outside mainland China in our research. First, in clinical studies, monitoring the dynamic changes of chest CT in patients with COVID-19 every day would be unrealistic and unnecessary. In a systematic review and meta-analysis on dynamic changes in COVID-19 images, Zhou et al. (97) revealed that, from 0 (negative CT) to 5 days (after firs positive CT), progressive deterioration of the lesions in the lungs, fluctuations of lesions (gradually improved and absorbed or gradually improved and absorbed after reaching the peak), seemingly did not achieve statistical significance. In a longitudinal study, Wang et al. (98) found that the common predominant pattern of chest CT had no changes on illness days 0–5 compared to days 12–17. For instance, the percentages of GGO and consolidation were 62%/23% on illness days 0–5 and 59%/24% on illness days 6–11, respectively. Second, chest CT changes in the course of COVID-19 were divided into two to three stages in most of the literature studies, including early stage, the progressive stage, and/or the severe stage, which are in accordance with the pathophysiological process of COVID-19 (48, 97, 98). Shi et al. (48) described and compared CT findings at different timepoints throughout the COVID-19 course and the grouping method was based on the interval between the onset of symptoms and the first CT scan: scans done before the onset of symptoms were group 1 and ≤ 1 week after the onset of symptoms were group 2. Zhou et al. (53) analyzed the CT features of COVID-19 and the dynamic changes of chest CT imaging features, and the course of the disease was divided into an early phase (≤ 7 days after the onset of symptoms) and an advanced phase (8–14 days after the onset of symptoms). Another study on the imaging features and the evolution of CT in patients with COVID-19 pneumonia showed that the early rapid progressive stage was 1–7 days from the onset of symptoms and the advanced stage with peak levels of abnormalities on CT was 8–14 days (99). Chang et al. (100) analyzed the CT findings of asymptomatic patients with COVID-19 and showed the differences between abnormal CT findings in the early phase (≤ 7 days after the onset of symptoms) and those in the advanced phase (8–14 days after the onset of symptoms).

Chest CT findings in COVID-19 are known to be different according to gender, age, and SARS-CoV-2 mutation (93, 101, 102). The reasons behind these disparities remain unclear; however, genetic, immunological, and social differences may be the essential contributing factors (103–105). However, it might seem unpractical and unnecessary to account for age, gender, and mutations based on the current meta-analysis. First, none of the included studies provided specific information on patient-level age, gender, and mutations, and the parameters could not be justified based solely on the data provided by the included studies. Second, no methodology that we have retrieved could justify these parameters in the type of meta-analysis and the methodology is imperfect and developing. Third, the existing literature found no significant difference in chest CT findings between men and women (106). Although age, gender, and mutations are important factors that evidently influence chest CT performance, last but not at least, the aim of this meta-analysis was to review the different CT features in patients with COVID-19 from within and outside mainland China to help radiologists and clinicians become more familiar with the disease, thus, including patients across all conditions might seem to be well-rounded.

Radiologists could accurately identify patients with COVID-19 positive using standard CT diagnostic procedures; however, there were obvious subjective factors and a lack of objective quantitative standards in the quantitative score of CT images to identify COVID-19 at the moment. Hence, the Radiological Society of North America (RSNA), the British Society of Thoracic Imaging (BSTI), the Dutch Radiological Society (COVID-19 Reporting and Data System, CO-RADS), etc., issued their expert consensus document to standardize COVID-19 CT findings and reports (107–109), among which RSNA is the most accepted structural reporting system (108, 110, 111). There is a bias in sensitivity values in most chest CT studies that do not include RSNA structural reporting system or others. Uysal et al. (112) reported that one-quarter of patients with asymptomatic COVID-19 had a normal chest CT. Kavak et al. (109) emphasized that false-negative patients should not be neglected in the RSNA and BSTI systems. Although the near perfect consistent and reproducible in the positive predictive value were observed in the system of RSNA and CO-RADS′ O′ Neill et al. (108) found that end-users preferred RSNA system for it's reporting language. From the experience of Falaschi et al. (113), the clinical and epidemiological features should be taken into account when using chest CT for the diagnosis of COVID-19. In addition, machine learning plays a pivotal role in the detection of COVID-19 pneumonia. For example, Herath et al. (114) developed an algorithm based on GGO with an accuracy of 92.8% and a precision of 0.931. On the one hand, studies that include the RSNA structural reporting system or machine learning algorithm should be included in this meta-analysis, and data for those that have and those that do not have the RSNA structural reporting system or machine learning algorithm should be presented separately. However, these parameters cannot be represented or extracted in our article. On the other hand, such worries that these data without the RSNA system are probably related to differences in outcome may not be necessary. Although without standards or quantitative scoring and with high subjectivity in these studies included in the manuscript, there are several reasons to think that the “qualitative evaluation” might not influence the differences in chest CT features between patients from within and outside mainland China: (1) though radiologists in the USA had minimal specific training to diagnose COVID-19 and Chinese radiologists practiced in an area with a relatively low prevalence of the disease, these radiologists could distinguish COVID-19 with high specificity and moderate sensitivity, which demonstrated that radiologists in and outside mainland China can identify COVID-19 with a high degree of agreement and high diagnosis accuracy (61). (2) Few literature studies reported that the corresponding standard or score (such as the CO-RADS lexicon) could help radiologists at different levels of experience to accurately distinguish patients with COVID-19 positive (115); however, the CT images included in this article were read by two or more senior radiologists with a host of experience, making the role of “standards” nonsignificant. (3) The sensitivity of manual qualitative analyses of chest CT for the diagnosis of COVID-19 within and outside mainland China is high (from 92 to 96%) (116–118), which is helpful for the early recognition of suspected cases, and the contribution of “standards” to sensitivity is really limited.

Our meta-analysis has several advantages. First, it was the first time to put forward the hierarchy of CT manifestation for patients in different regions, which could guide the clinical work. Second, a wide range of search strategies but strict inclusion criteria were used to minimize the possibility of publication bias. Third, compared to prior studies, we excluded non-English literature to ensure the accuracy of the results. Finally, the included studies were conducted in different countries or regions, which made the results more representative.

Nevertheless, our present meta-analysis inevitably has some disadvantages and limitations. First, all of the included studies were comparative trials or cohort studies that did not provide the powerful statistical power that a randomized controlled trial could do. Second, neither the different degrees of severity for patients with COVID-19 (asymptomatic, mild, moderate, or severe) nor possible comorbidities or chronic diseases could be distinguished from most of the included studies. Third, patients outside mainland China were fewer than those inside, which may bias their estimates. Finally, some of the subgroup analyses for patients within mainland China showed some publication biases, which might affect the accuracy of the results. Future studies into COVID-19 should focus on exploring the mechanism of the significant differences in chest CT in different regions, which could provide more experience and evidence regarding COVID-19 diagnosis and management.

## Conclusion

In conclusion, there is no doubt that chest CT plays an important role in the detection of patients with suspected COVID-19; however, the CT patterns of patients in mainland China cannot reflect those of the patients outside mainland China. Given substantial differences in chest CT between patients from within and outside mainland China and the low specificity in differentiating cases of viral pneumonia, radiologists and clinicians should be familiar with various CT manifestations suggestive of COVID-19 in different regions and should be carefully adjudicated.

## Data availability statement

The original contributions presented in the study are included in the article/Supplementary material, further inquiries can be directed to the corresponding author/s.

## Author contributions

NH and KW designed this study. LW, ML, BX, and LH conducted literature searches and screening, literature data extraction, and statistical analysis. MW, MG, and SL checked the extracted data. ML, BX, and MG wrote the first draft. RZ, KW, and NH corrected the manuscript and supervised the conduct of the study. All authors have read and approved the final submitted version.

## Funding

This work was supported by grants from the Science and Technology Development Project of Medical and Health of Shandong Province (Nos. 202010000131 and 202104070065) and the Key Research and Development Project of Zibo (Policy Guidance Program, No. 2020ZC010048).

## Conflict of interest

The authors declare that the research was conducted in the absence of any commercial or financial relationships that could be construed as a potential conflict of interest.

## Publisher's note

All claims expressed in this article are solely those of the authors and do not necessarily represent those of their affiliated organizations, or those of the publisher, the editors and the reviewers. Any product that may be evaluated in this article, or claim that may be made by its manufacturer, is not guaranteed or endorsed by the publisher.

## Supplementary material

Supplementary Figure S1 Methodological evaluation according to Quality Assessment of Diagnostic Accuracy Studies (QUADAS) of the included studies by grouped bar charts, proportion of studies with low, high, or unclear risk of bias **(A)**, and concerns regarding applicability **(B)**.

Supplementary Figure S2 Forest plots of bilateral lung infection manifested by chest computerized tomography (CT) for patients with COVID-19. Forest plots show the transformed incidence rate of bilateral lung involvement in all patients **(A)**, patients in mainland China **(B)**, and patients outside mainland China **(C)**. COVID-19: coronavirus disease 2019; CI, confidence interval.

Supplementary Figure S3 Forest plots of subgroup analyses of typical CT imaging appearances by chest CT for overall patients with COVID-19: GGO **(A)**, consolidation **(B)**, crazy-paving sign **(C)**, mixed GGO and consolidation **(D)**, air bronchogram **(E)**, nodules **(F)**, vascular engorgement **(G)**, bronchial wall thickening **(H)**, septal thickening **(I)**, and pleural effusion **(J)**. COVID-19: coronavirus disease 2019; CI, confidence interval; GGO, ground-glass opacities.

Supplementary Figure S4 Forest plots of subgroup analyses of typical CT imaging appearances by chest CT for patients with COVID-19 within mainland China: GGO **(A)**, consolidation **(B)**, crazy-paving sign **(C)**, mixed GGO and consolidation **(D)**, air bronchogram **(E)**, nodules **(F)**, vascular engorgement **(G)**, bronchial wall thickening **(H)**, septal thickening **(I)**, and pleural effusion **(J)**. COVID-19: coronavirus disease 2019; CI, confidence interval; GGO: ground-glass opacities.

Supplementary Figure S5 Forest plots of subgroup analyses of typical CT imaging appearances by chest CT for patients with COVID-19 outside mainland China: GGO **(A)**, consolidation **(B)**, crazy-paving sign **(C)**, mixed GGO and consolidation **(D)**, air bronchogram **(E)**, nodules **(F)**, vascular engorgement **(G)**, bronchial wall thickening **(H)**, septal thickening **(I)**, and pleural effusion **(J)**. COVID-19: coronavirus disease 2019; CI, confidence interval; GGO: ground-glass opacities.

Supplementary Figure S6 Forest plots of subgroup analyses of typical CT imaging appearances by chest CT for overall patients with COVID-19: halo sign **(A)**, linear opacities **(B)**, lymphadenopathy **(C)**, and pleural thickening **(D)**. COVID-19: coronavirus disease 2019; CI, confidence interval; GGO: ground-glass opacities.

Supplementary Table S1 The characteristics of the included studies.

Supplementary Table S2 Risk of bias assessment of the included studies according to the Quality Assessment of Diagnostic Accuracy Studies (QUADAS) scale.

## Abbreviations

COVID-19: Coronavirus disease 2019
SARS-CoV-2: Severe acute respiratory syndrome coronavirus 2
RT-PCR: Reverse transcription-polymerase chain reaction
CT: Computerized tomography
PRISMA: Preferred Reporting Items for Systematic Reviews and Meta-Analyses guidelines
GGO: Ground-glass opacities
QUADAS: Quality assessment of diagnostic accuracy studies
HRCT: Ultra-high-resolution CT
HD-DECT: High-dose dual-energy acquisition CT
LDCT: Low-dose CT
CI: Confidence interval
SARS: Severe acute respiratory syndrome
MERS: Middle East respiratory syndrome
ACE2: Angiotensin-converting enzyme-2
ARBs: Angiotensin receptor blockers
ALI: Acute lung injury
RSNA: the Radiological Society of North America
BSTI: the British Society of Thoracic Imaging
CO-RADS: the Dutch Radiological Society (COVID-19 Reporting and Data System).
