# Supplementary material for: Do COVID-19 CT features vary between patients from within and outside mainland China? Findings from a meta-analysis
Source: Front Public Health. 2022 Oct 14;10:939095. doi: 10.3389/fpubh.2022.939095 (PMC9616120; doi:10.3389/fpubh.2022.939095)
Supplement: Supplementary Figure S1 — Methodological evaluation according to Quality Assessment of Diagnostic Accuracy Studies (QUADAS) of the included studies by grouped bar charts, proportion of studies with low, high, or unclear risk of bias (A), and concerns regarding applicability (B). [file Data_Sheet_1.pdf]

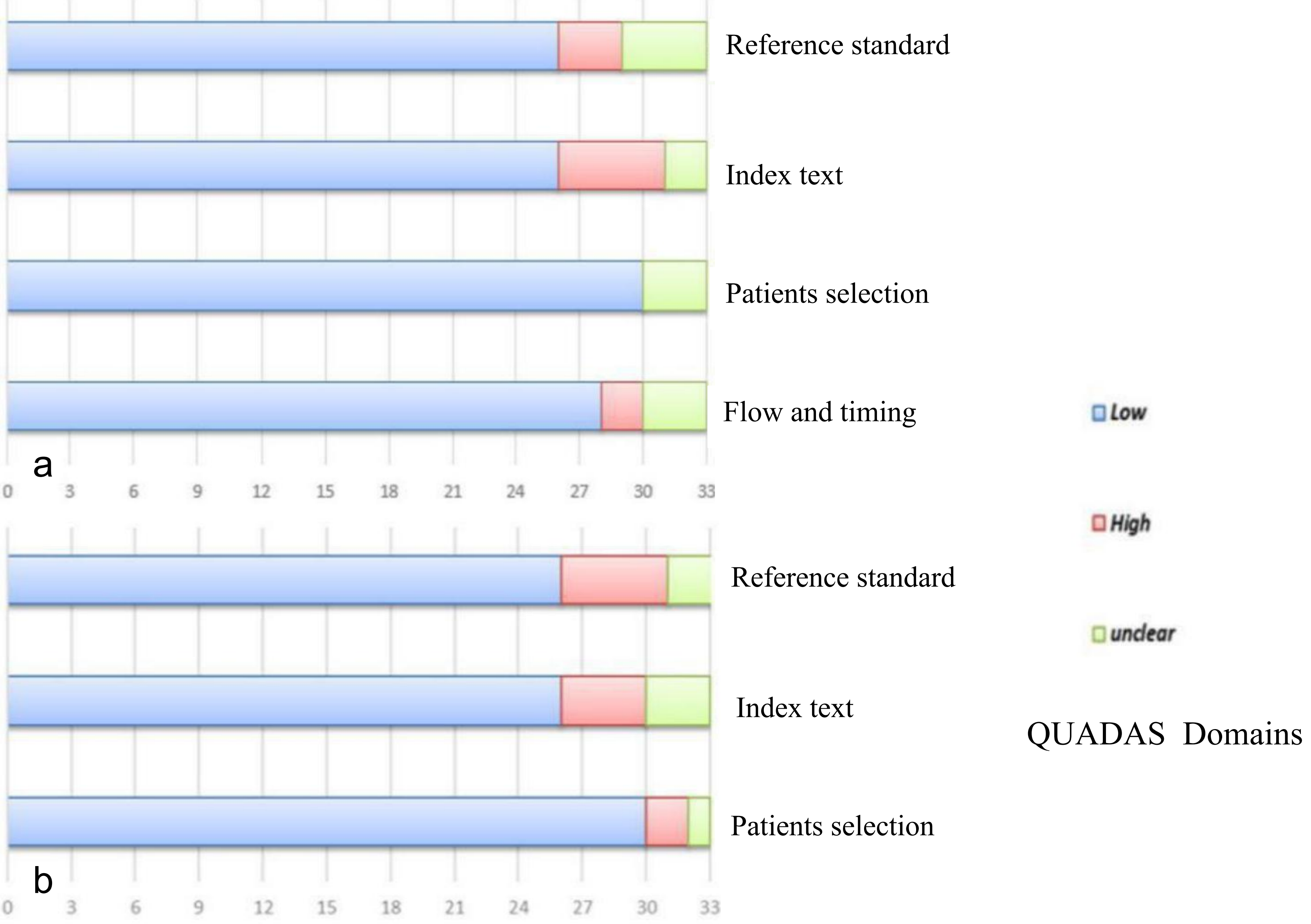

Supplement figure 1: Methodological evaluation according to QUADAS-2 of the included studies by grouped bar charts , Proportion of studies with low, high or unclear RISk of Bias (a), and Concerns regarding Applicability (b).
