# Supplementary figures and images for: Do COVID-19 CT features vary between patients from within and outside mainland China? Findings from a meta-analysis
Source: Front Public Health. 2022 Oct 14;10:939095. doi: 10.3389/fpubh.2022.939095 (PMC9616120; doi:10.3389/fpubh.2022.939095)

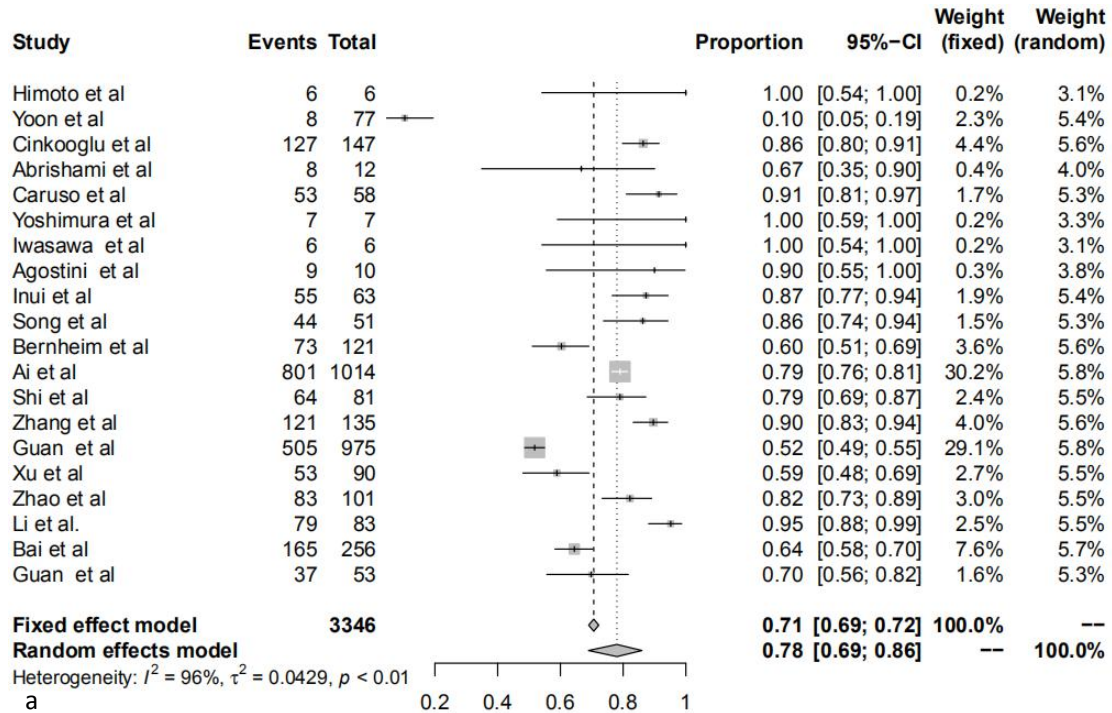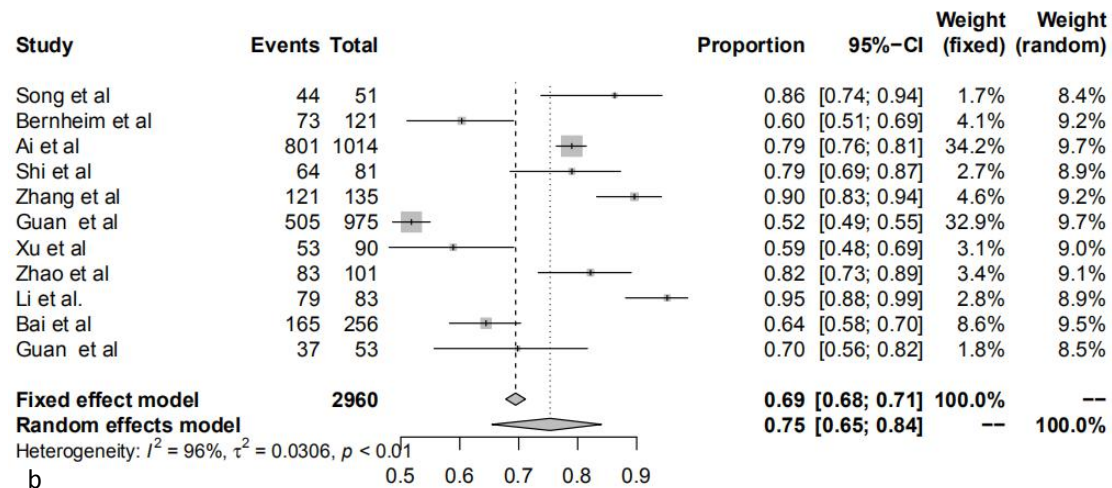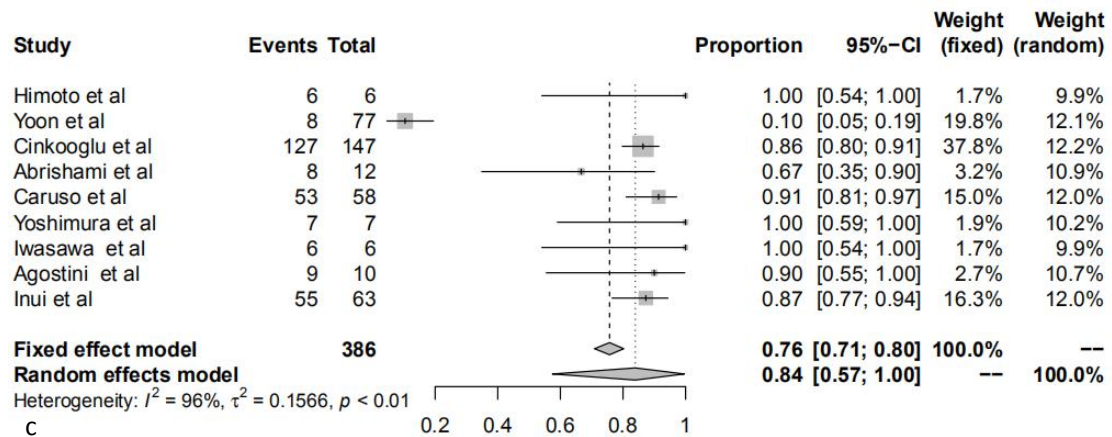

Supplement: Supplementary Figure S2 — Forest plots of bilateral lung infection manifested by chest computerized tomography (CT) for patients with COVID-19. Forest plots show the transformed incidence rate of bilateral lung involvement in all patients (A), patients in mainland China (B), and patients outside mainland China (C). COVID-19: coronavirus disease 2019; CI, confidence interval. [file Data_Sheet_2.pdf]

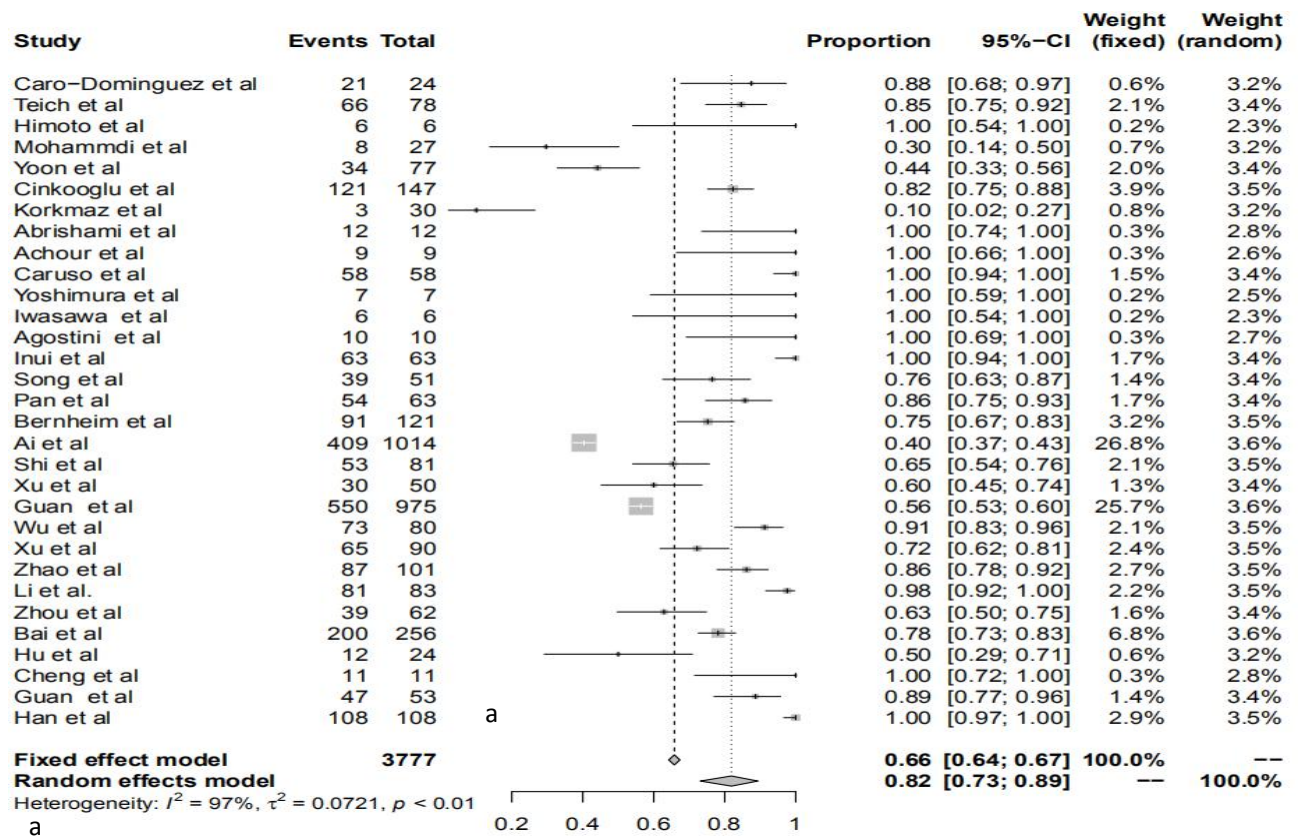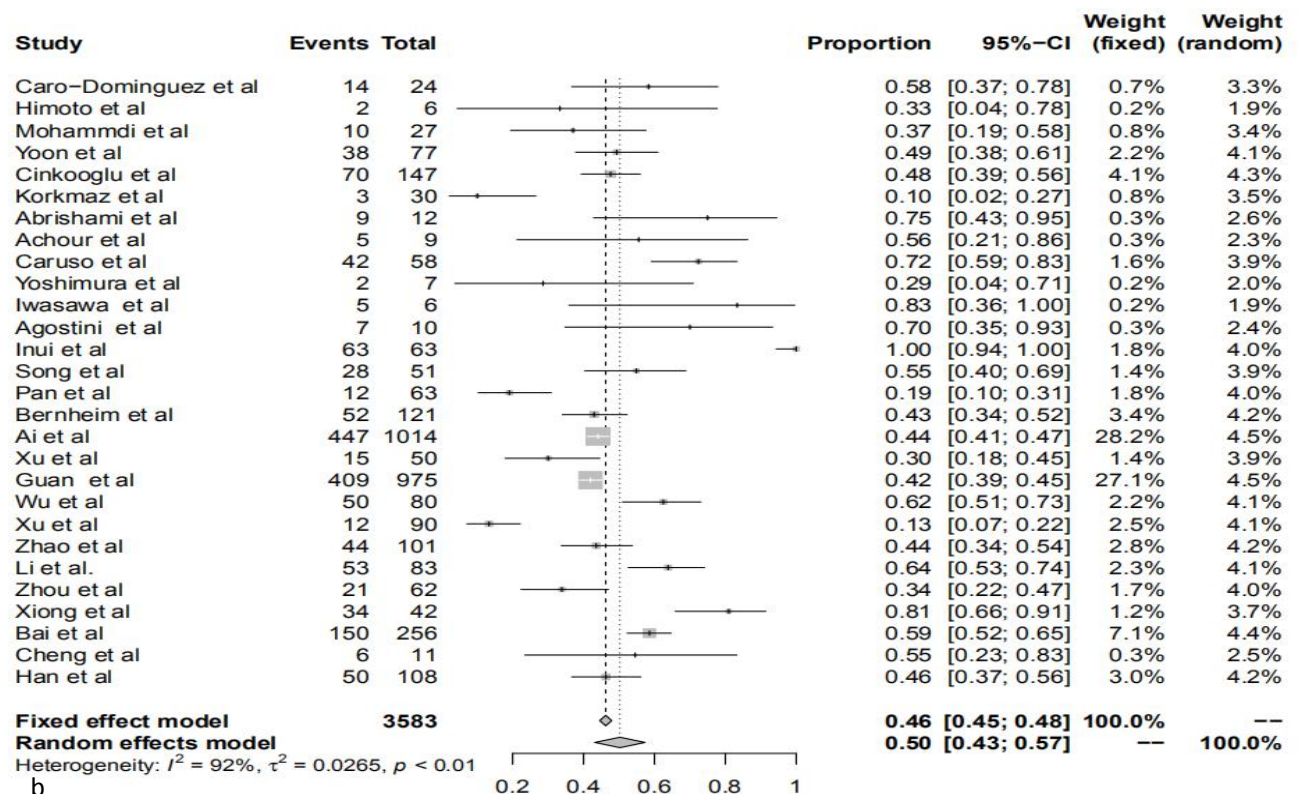

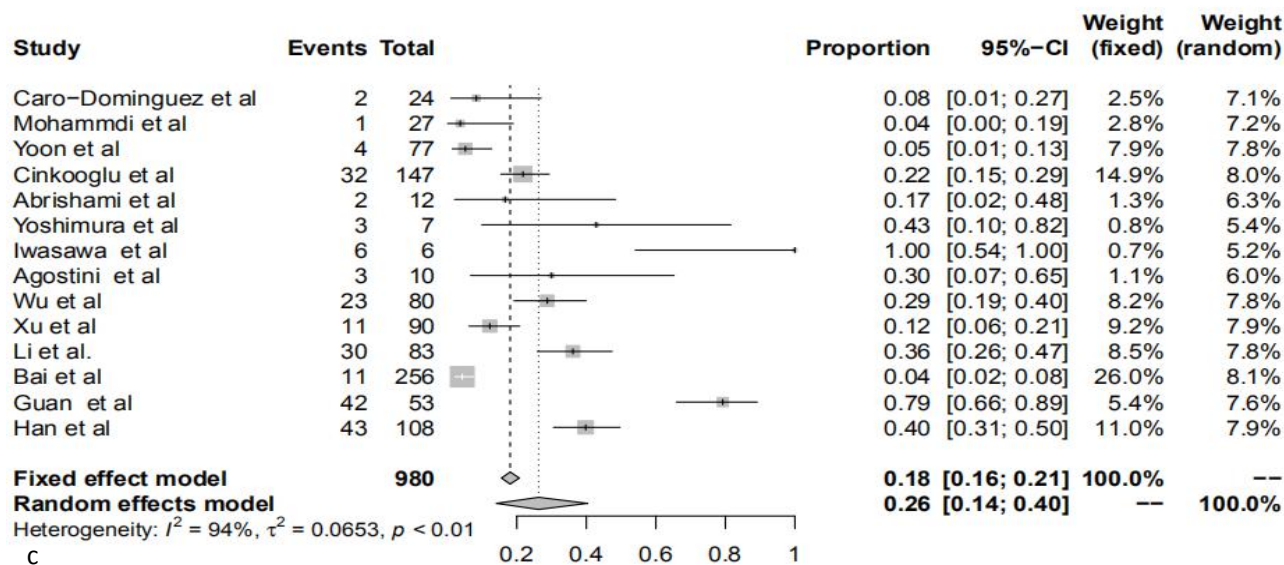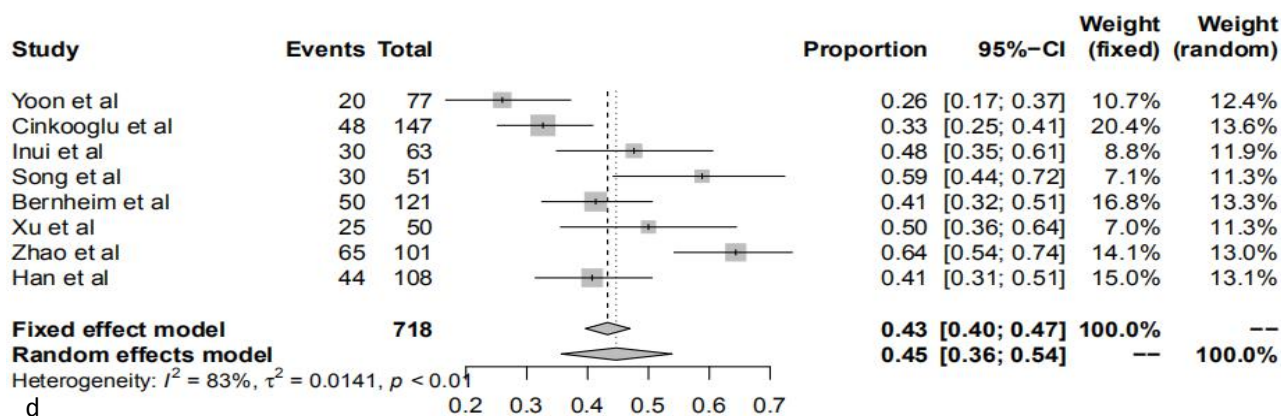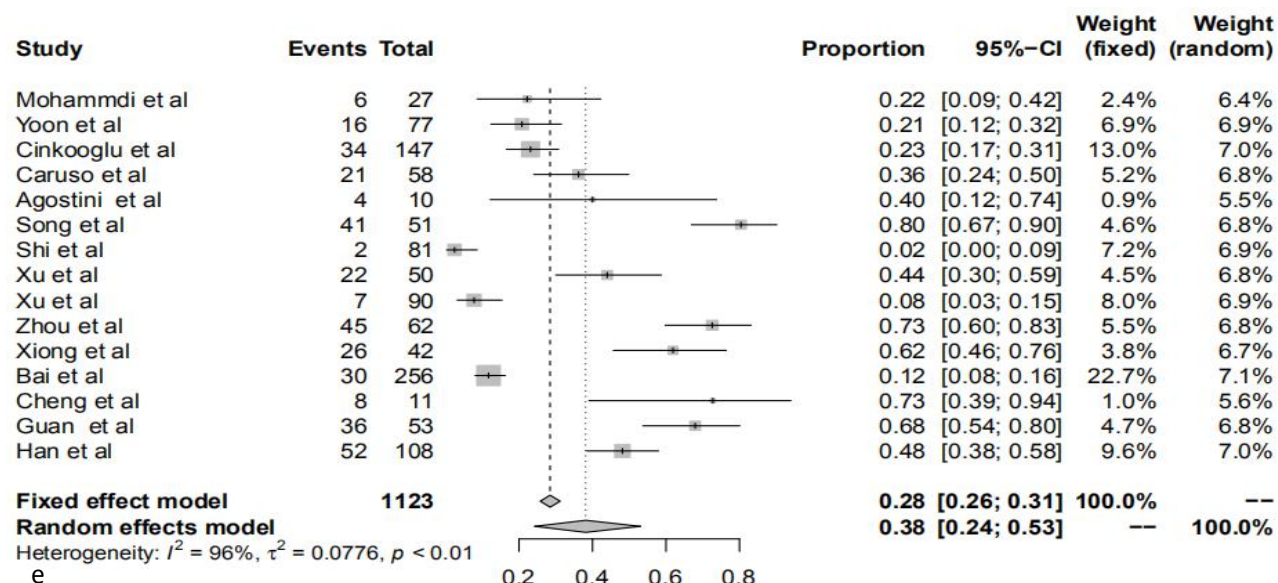

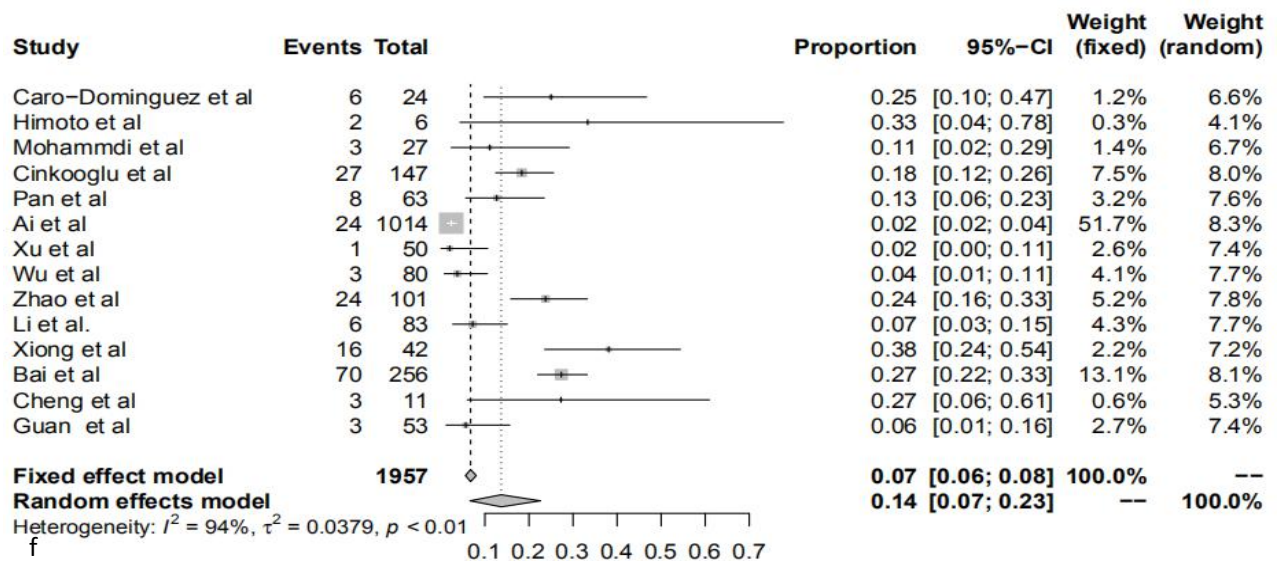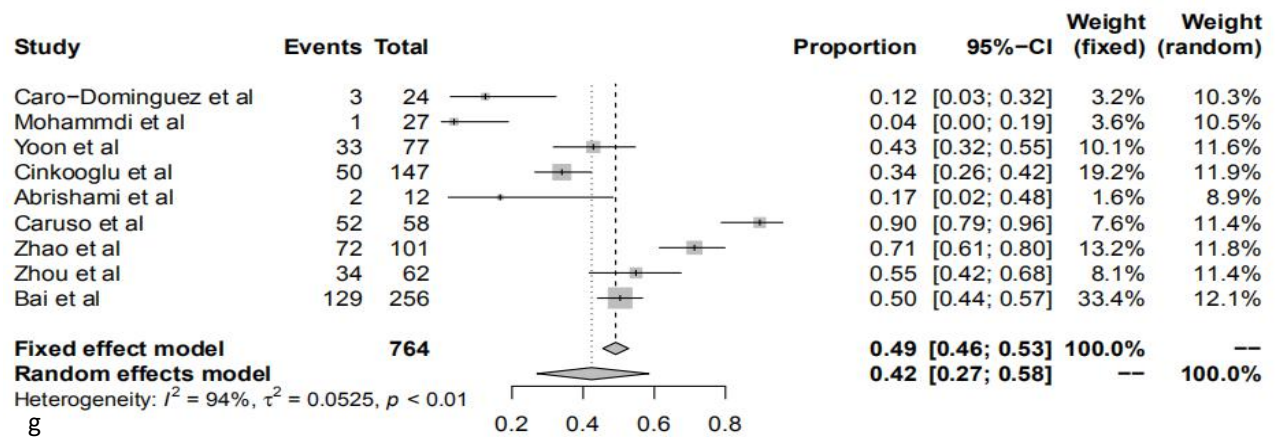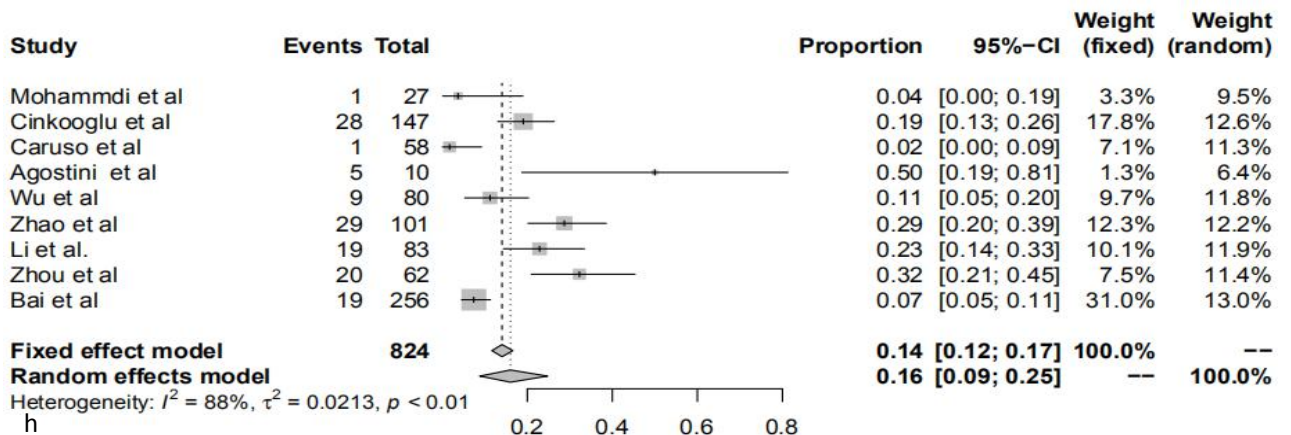

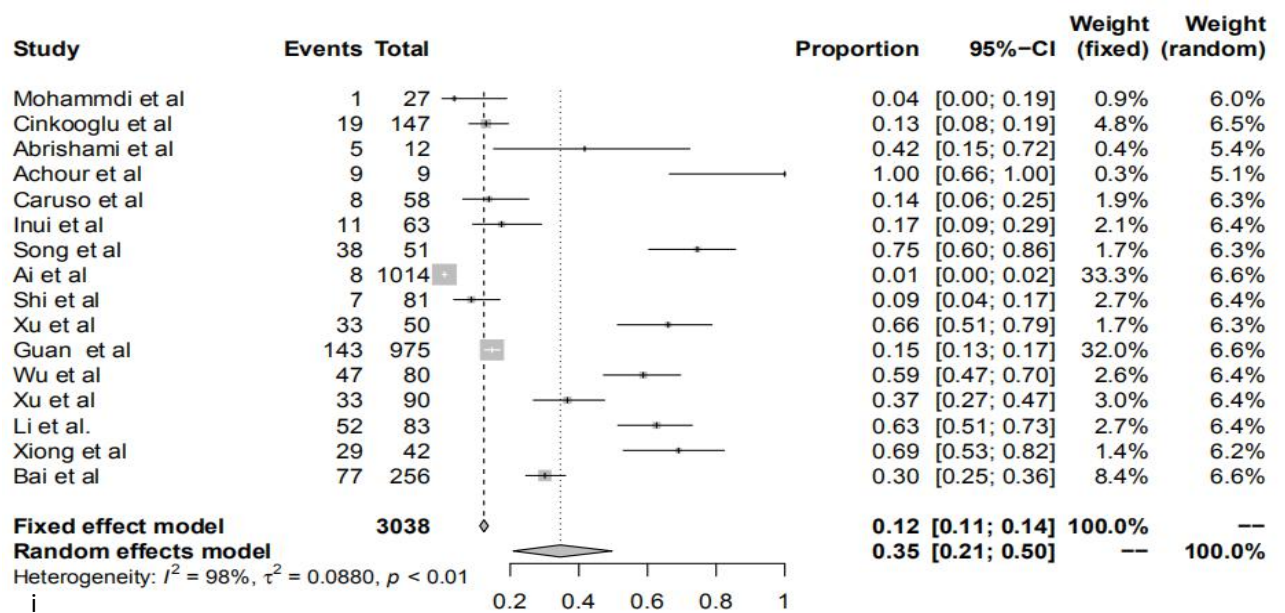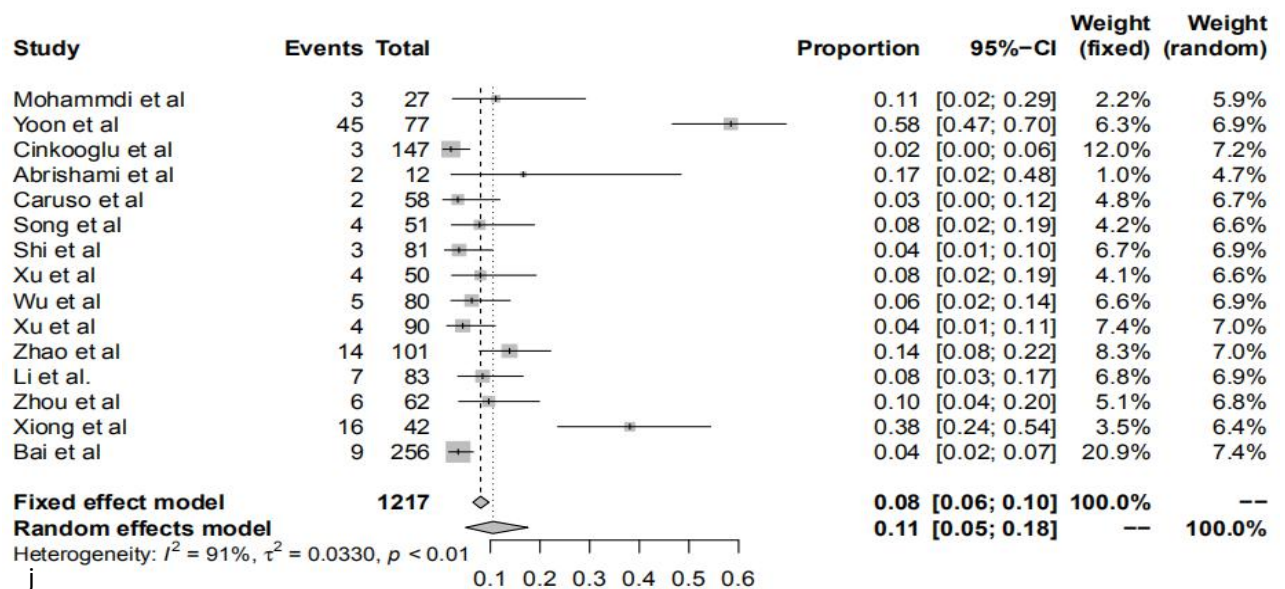

Supplement: Supplementary Figure S3 — Forest plots of subgroup analyses of typical CT imaging appearances by chest CT for overall patients with COVID-19: GGO (A), consolidation (B), crazy-paving sign (C), mixed GGO and consolidation (D), air bronchogram (E), nodules (F), vascular engorgement (G), bronchial wall thickening (H), septal thickening (I), and pleural effusion (J). COVID-19: coronavirus disease 2019; CI, confidence interval; GGO, ground-glass opacities. [file Data_Sheet_3.pdf]

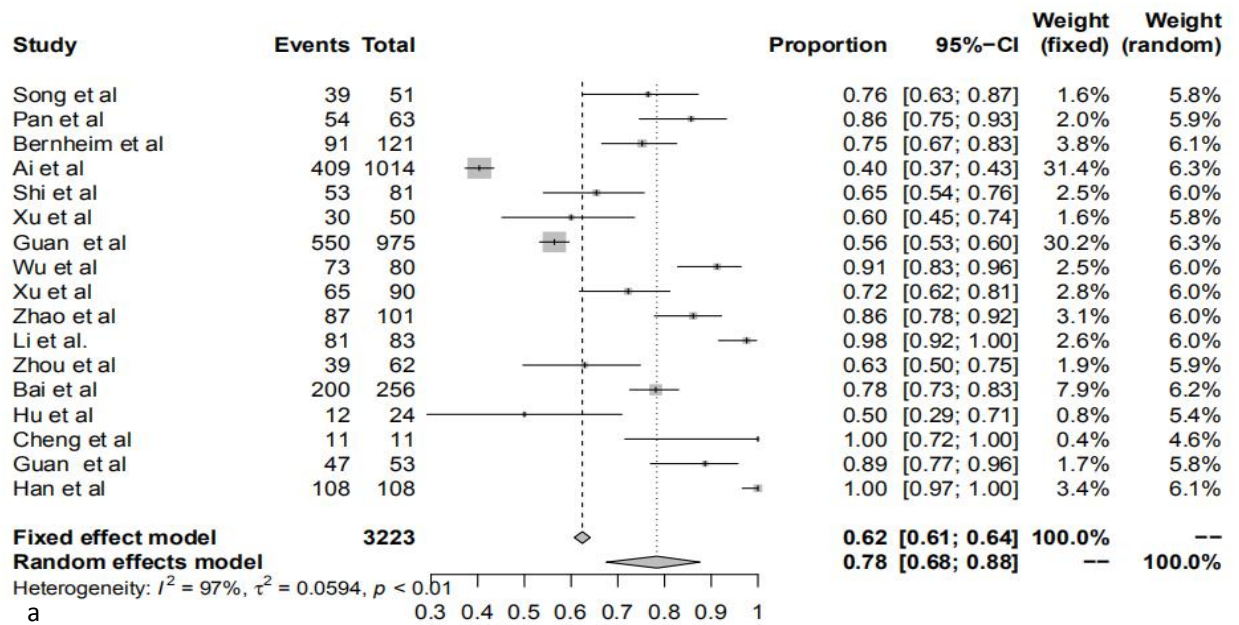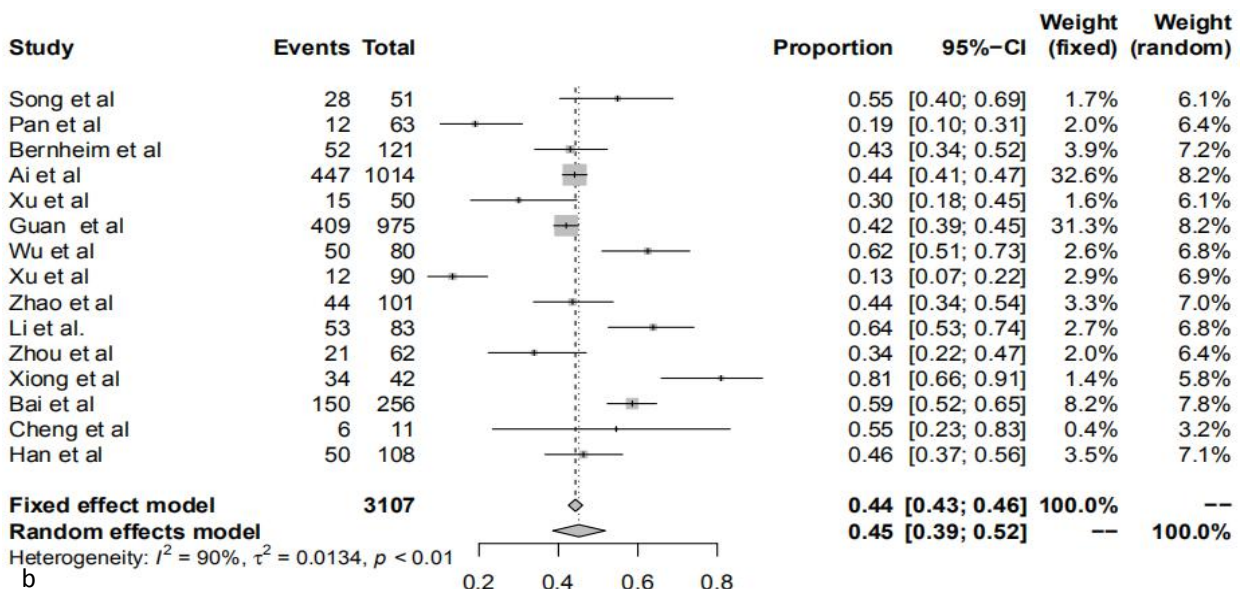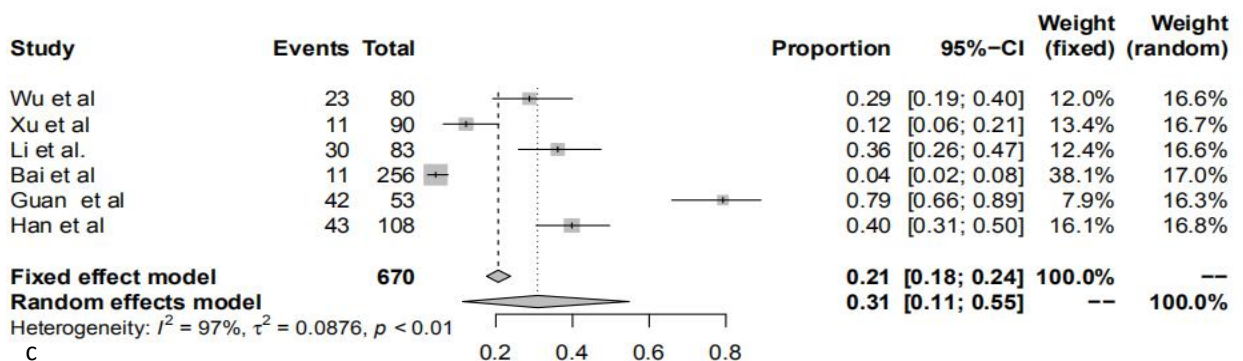

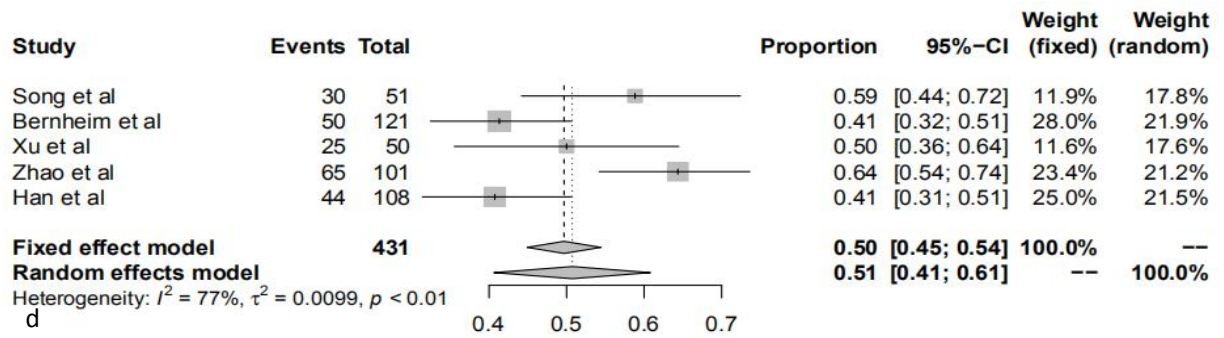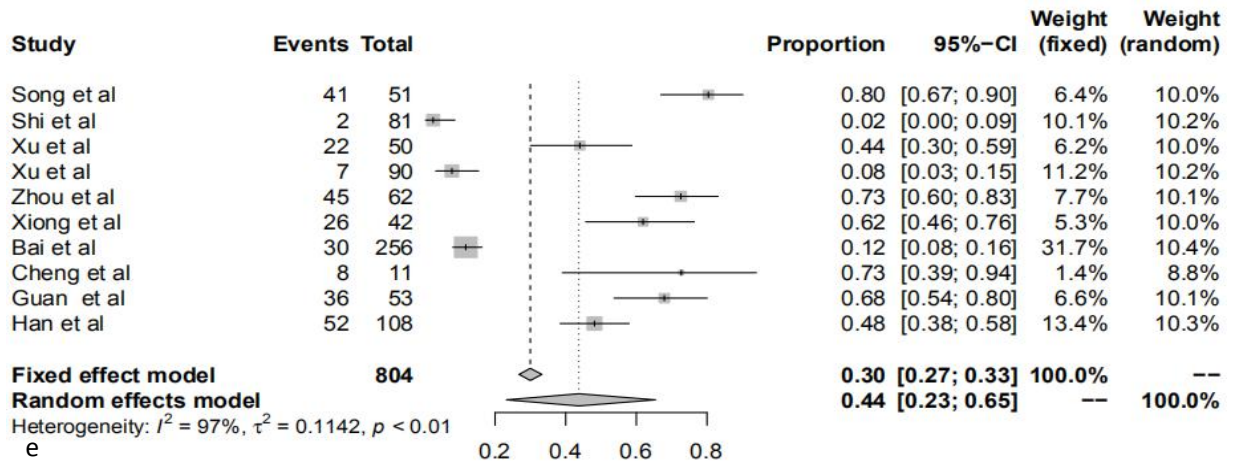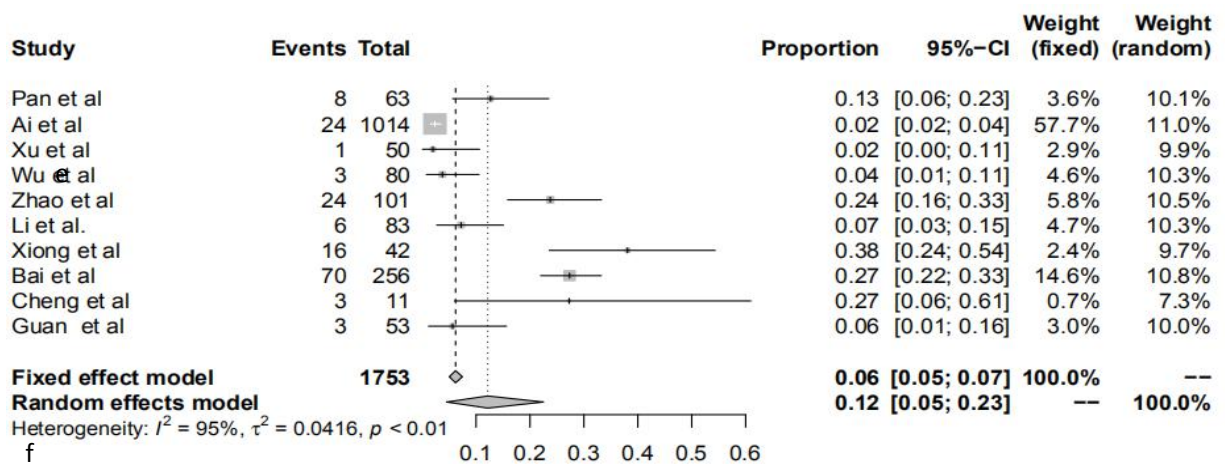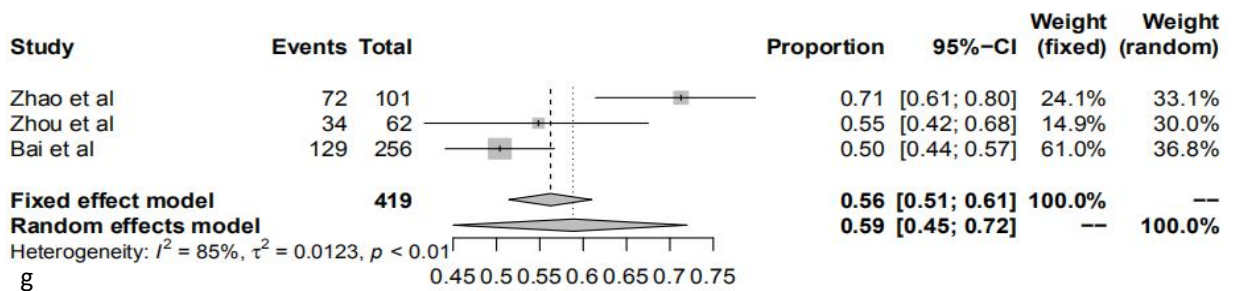

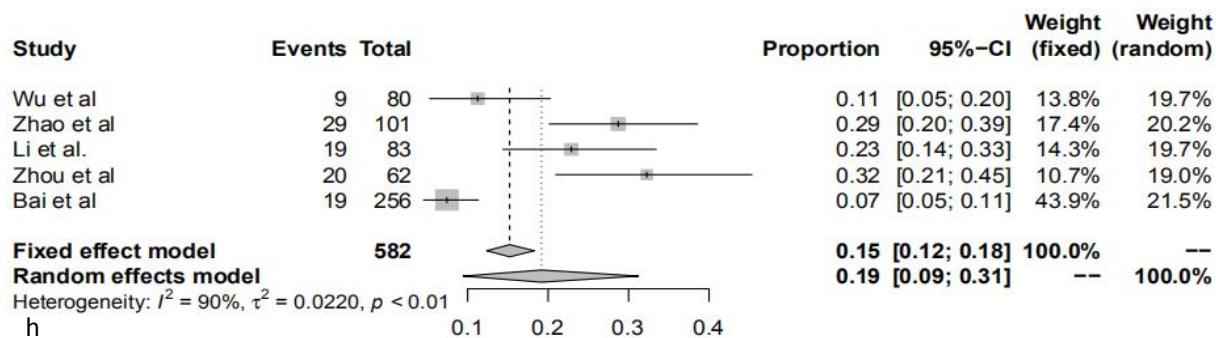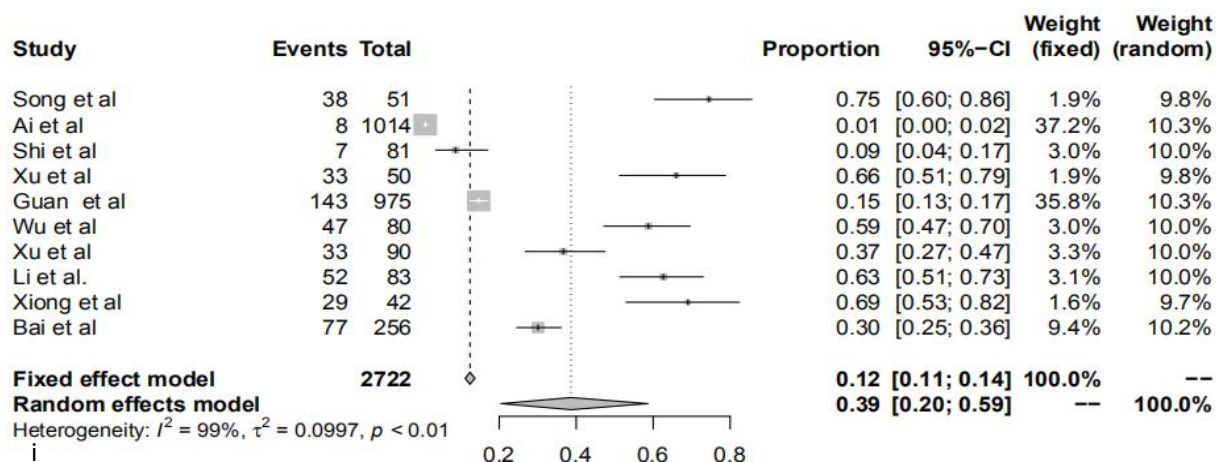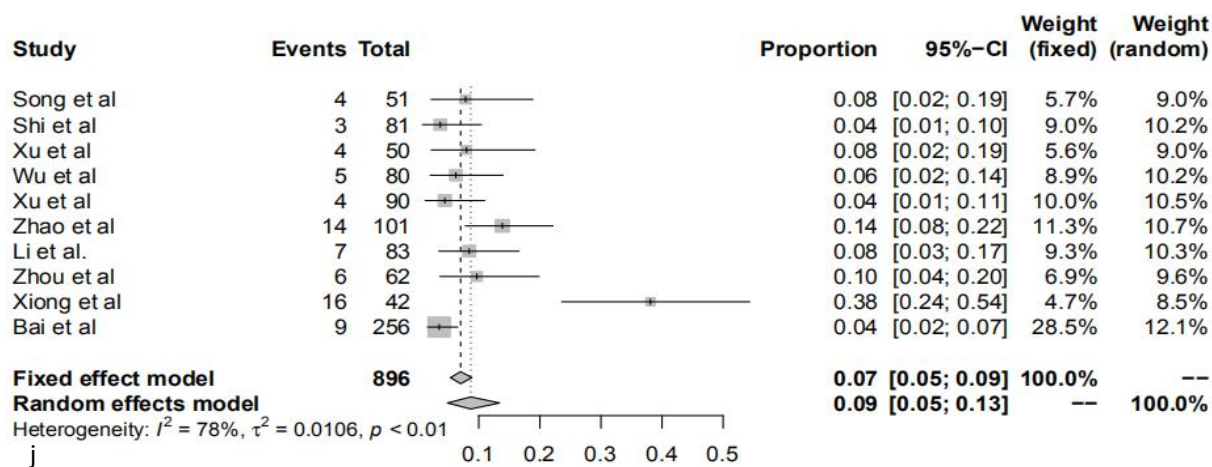

Supplement: Supplementary Figure S4 — Forest plots of subgroup analyses of typical CT imaging appearances by chest CT for patients with COVID-19 within mainland China: GGO (A), consolidation (B), crazy-paving sign (C), mixed GGO and consolidation (D), air bronchogram (E), nodules (F), vascular engorgement (G), bronchial wall thickening (H), septal thickening (I), and pleural effusion (J). COVID-19: coronavirus disease 2019; CI, confidence interval; GGO: ground-glass opacities. [file Data_Sheet_4.pdf]

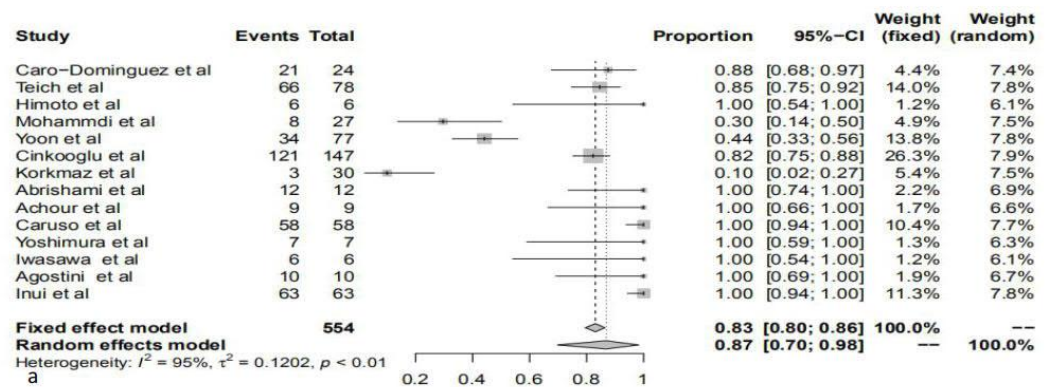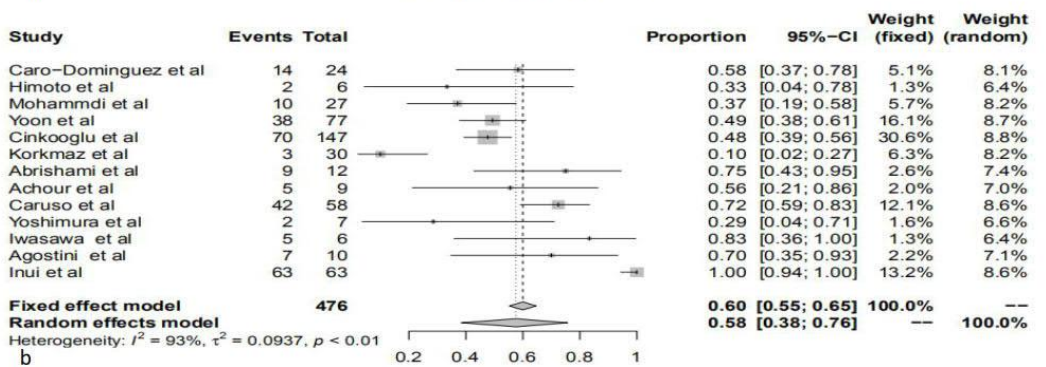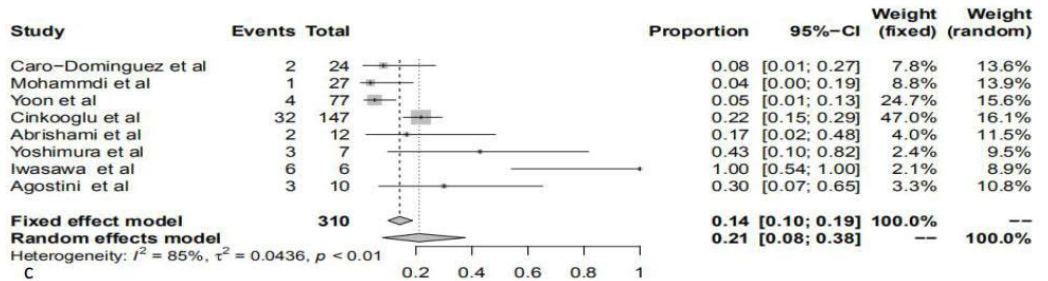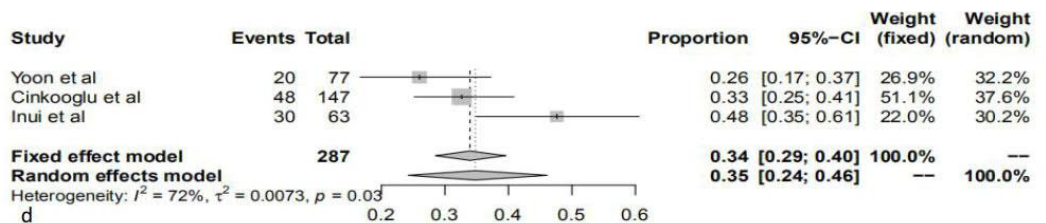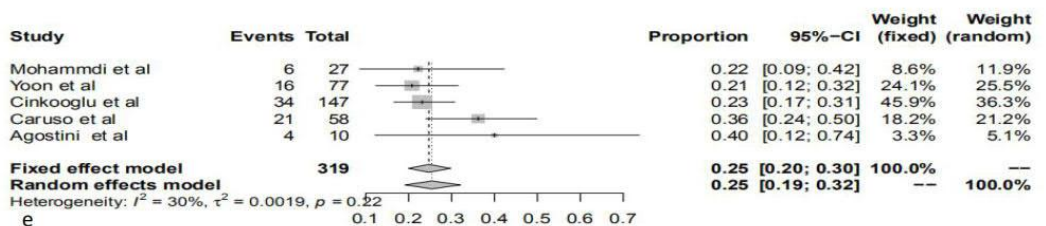

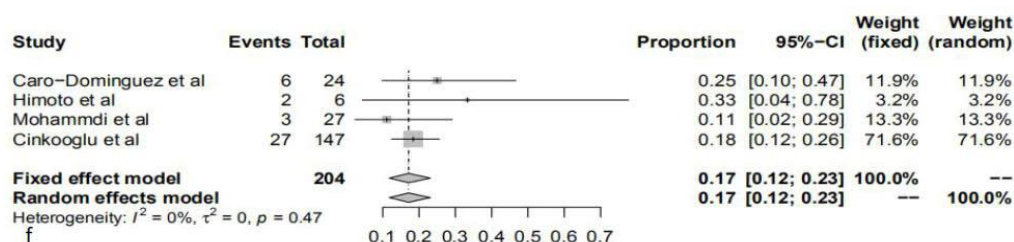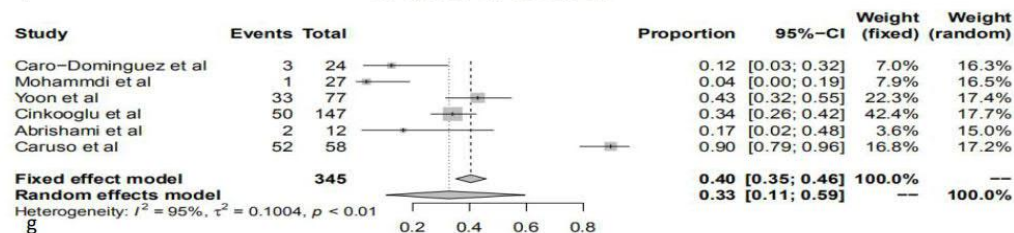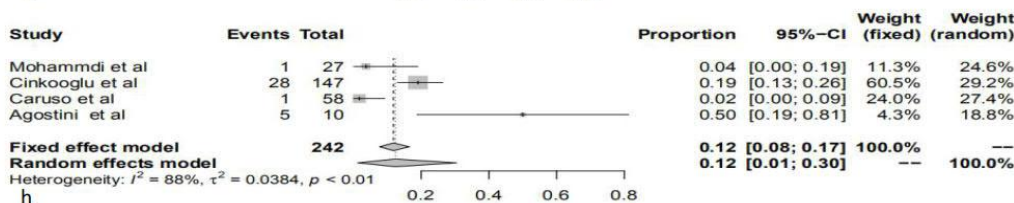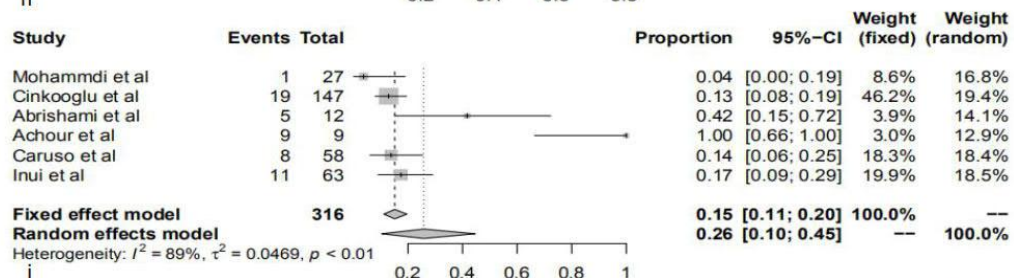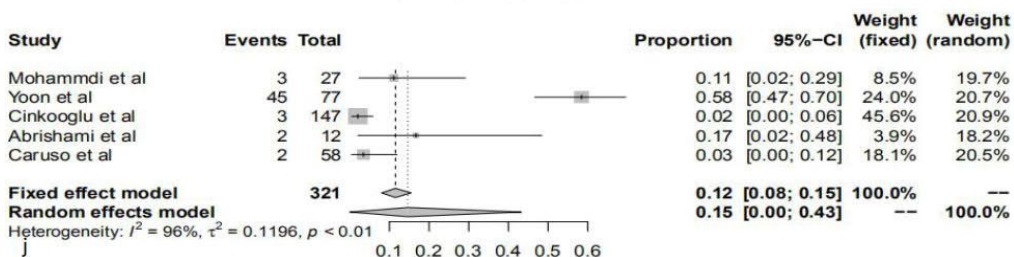

Supplement: Supplementary Figure S5 — Forest plots of subgroup analyses of typical CT imaging appearances by chest CT for patients with COVID-19 outside mainland China: GGO (A), consolidation (B), crazy-paving sign (C), mixed GGO and consolidation (D), air bronchogram (E), nodules (F), vascular engorgement (G), bronchial wall thickening (H), septal thickening (I), and pleural effusion (J). COVID-19: coronavirus disease 2019; CI, confidence interval; GGO: ground-glass opacities. [file Data_Sheet_5.pdf]

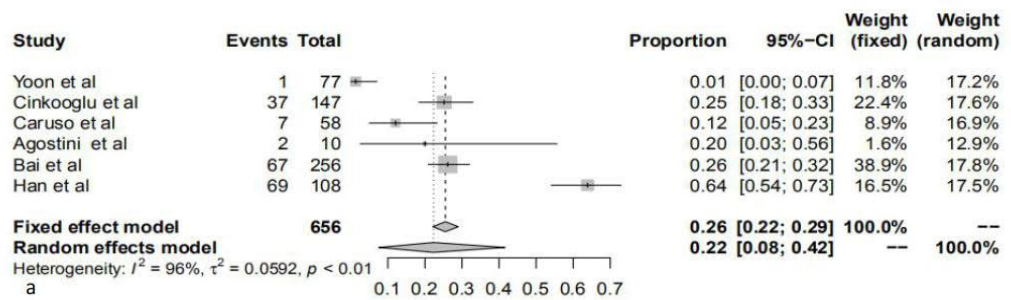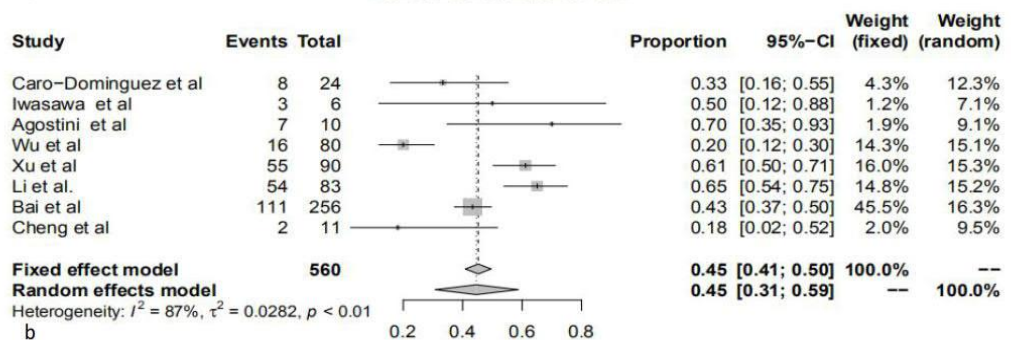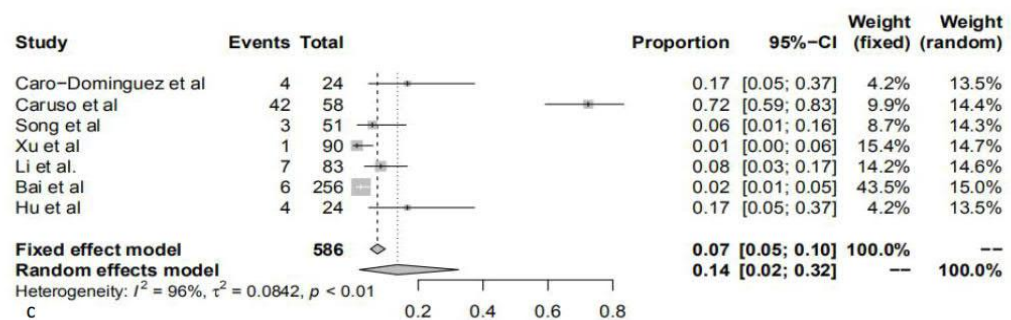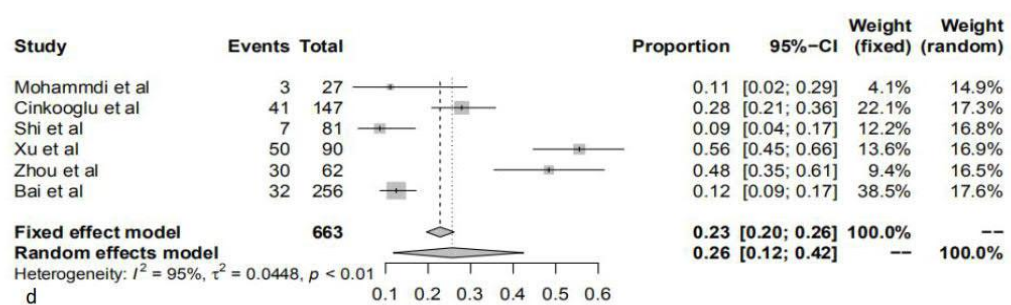

Supplement: Supplementary Figure S6 — Forest plots of subgroup analyses of typical CT imaging appearances by chest CT for overall patients with COVID-19: halo sign (A), linear opacities (B), lymphadenopathy (C), and pleural thickening (D). COVID-19: coronavirus disease 2019; CI, confidence interval; GGO: ground-glass opacities. [file Data_Sheet_6.pdf]
