# Supplementary material for: Do COVID-19 CT features vary between patients from within and outside mainland China? Findings from a meta-analysis
Source: Front Public Health. 2022 Oct 14;10:939095. doi: 10.3389/fpubh.2022.939095 (PMC9616120; doi:10.3389/fpubh.2022.939095)
Supplement: Supplementary Table S1 — The characteristics of the included studies. [file Table_1.docx]

Supplement table 1: The characteristics of the included studies

| Study | Countries/Regions | Sample Size | Diagnostic criteria | Experimental design method | Mean /  Median Age | Gender(Male/ Female) | Special CT | CT  Abnormal | CT Imaging  Manifestations |
| --- | --- | --- | --- | --- | --- | --- | --- | --- | --- |
|  |  |  |  |  |  |  |  |  |  |
| Caro-Dominguez et al. [32] | European countries,Iran,Mexico  and HK | 81(24 with chest  CT ) | RT-PCR | Retrospective observational study | 13.5 | 12/12 | Without mention | 22 | GGO,Consolidations, Linear opacities, Nodules,Tree in bud appearance, Lymphadenopathy, Vascular engorgement,Crazy paving pattern |
| Himoto et al. [33] | Japan | 21(6 diagnosed COVID-19) | RT-PCR | Retrospective cohort study | 58.5 | 5/1 | Without mention | 6 | GGO ,Consolidations ,opacities4,Pulmonary emphysema, Pulmonary nodules |
| Mohammdi et al. [34] | Iran | 27 | RT-PCR | Retrospective cohort study | 4.7 | 10/17 | Without mention | 18 | Ground-glass opacities , Air-bronchogram , Consolidation, Cystic lesions ,Reticular lesion ,Vascular thickening , Crazy paving ,Nodules ,Septal thickening , Bronchial wall thickening1, Pleural efusion ,Pleural thickening |
| Yoon et al. [35] | Korean | 9(77 lung lesions) | RT-PCR | Retrospective cohort study | 54 | 4/5 | Without mention | 54 | GGO,Consolidation ,Crazy-paving appearance ,Mixed GGO and consolidation |
| *Çinkooğlu* et al. [36] | Turkey | 185(147 diagnosed COVID-19) | RT-PCR | Retrospective cohort study | 48.7 | 87/98 | HRCT | 109 | Pure GGO , GGO with consolidation, Crazy paving pattern ,  Pure consolidation , Solid nodules |
|  |  |  |  |  |  |  |  |  |  |
| Abrishami et al.[37] | Iran | 12 | RT-PCR and | Retrospective cohort study | 47.7 | 9/3 | Without mention | 12 | Ground glass opacity , Consolidation , Interlobular septal thickening, Dilated small vessels in the lesion , Crazy-paving , Pleural effusion, Pericardial effusion |
| Achour et al. [38] | Tunisia | 542(9diagnosed COVID-19) | RT-PCR  and Chest CT | Retrospective cohort study | 45 | 357/185 | Without mention | 9 | Ground-glass opacity , Consolidation |
| Caruso et al. [39] | Italy | 158（58with chest  CT and postive PCR） | RT-PCR and Chest CT | Prospective study | 57 | 83/75 | Without mention | 58 | Ground Glass Opacity (GGO) , Consolidation , Lymphadenopathy ,  Bronchiectasis ,Air Bronchogram , Pulmonary nodules surrounded by GGO |
|  |  |  |  |  |  |  |  |  |  |
| Iwasawa et al. [40] | Japan | 6 | RT-PCR | Retrospective cohort study | 69.2 | 2/4 | U-HR-CT | 6 | GGO,Consolidation ,Linear opacities ,Crazy paving pattern |
| Agostini et al. [41] | Italy | 10 | RT-PCR | Prospective cohort study | 53 | 3/7 | HD-DECT /LDCT | 10 | Ground glass opacities ,Linear opacities , “Crazy-Paving” pattern , “Reverse Halo” Sign ,Bronchial Wall Thickening , Bronchiectasis , Bilateral lung involvement |
| Inui et al. [42] | Japan | 104（63 with lung opacities） | RT-PCR | Prospective cohort study | 61.4 | 54/50 | Without mention | 63 | Lung opacities ,Airway abnormalities ,Emphysema ,Pulmonary fibrosis |
| Teich et al. [43] | Brazil | 510（78 with chest  CT） | RT-PCR | Retrospective cohort study | 39.9 | 290/220 | Without mention | 73 | GGO ,Local patchy shadowing ,Bilateral patchy shadowing ,Interstitial abnormalities |
| Korkmaz et al. [44] | Turkey | 81(30with chest  CT) | RT-PCR | Retrospective cohort study | 9.5 | 48/33 | Without mention | 6 | Consolidation ,Ground-glass opacities |
| Yoshimura et al. [45] | Japan,USA,Australia,  HK,Taiwan | 17（7with chest  CT） | RT-PCR | Retrospective cohort study | 69 | 8/9 | Without mention | 7 | Bilateral lesions ,Ground-glass opacities ,Peripheral dominance ,Reticular pattern , Crazy paving pattern , Consolidations |
| Wu et al. [16] | China | 80 | Chest CT | retrospective study | 46.1 | 39/41 | Without mention | 55 | Bilateral lung |
| Ai et al. [17] | China | 1,014 | RT-PCR | retrospective study | 51 | 467/547 | Thin-section  Chest CT | 888 | Bilateral lung, GGO, consolidation, interlobular  septal thickening |
|  |  |  |  |  |  |  |  |  |  |
| Pan et al. [46] | China | 63 | Chest CT | Retrospective, cross section study | 44.9 | 33/30 | Thin-section  Chest CT | 63 | GGO, consolidation |
| Bernheim  et al. [47] | China | 121 | RT-PCR | retrospective study | 45.3 | 61/60 | 99 with thin-section/22 with  conventional CT | 94 | GGO, consolidation, crazy paving pattern, pleural effusion, bronchiectasis, lymphadenopathy |
| Shi et al. [48] | China | 81 | next-generation sequencing or RT-PCR | retrospective study | 49.5 | 42/39 | Thin-section  Chest CT | 81 | Bilateral lung, GGO, consolidation, interlobular  septal thickening, adjacent pleura thickening, air bronchogram, pleural effusion, bronchiectasis, lymphadenopathy |
| Xu et al. [49] | China | 50 | Chest CT | retrospective study | 43.9 | 29/21 | Thin-section  Chest CT | 41 | GGO, consolidation, interlobular septal thickening, air bronchogram, pleural effusion, |
| Xu et al. [50] | China | 90 | RT-PCR | retrospective study | 50 | 39/51 | Thin-section  Chest CT | 69 | Bilateral lung, GGO, consolidation, crazy paving  pattern, interlobular septal thickening, air  bronchogram, adjacent pleura thickening, pleural effusion, pericardial effusion,  lymphadenopathy, |
| Zhao et al. [51] | China | 101 | RT-PCR | retrospective study | 44.4 | 87/39 | Without mention | 93 | GGO,Consolidation Mixed ground-glass opacities and consolidation,Centrilobular nodules,  Architectural distortion,Bronchial wall thickening,Traction bronchiectasis,Intrathoracic lymph node enlargement ,Vascular enlargement,Pleural effusions |
| Li et al. [52] | China | 83 | Diagnosis and Treatment of Novel Coronavirus Pneumonia (Fifth Trial  Version) of China | retrospective study | 45.5 | 44/39 | Without mention | 83 | GGO, Linear opacities ,Consolidation, Interlobular septal thickening, Crazy-paving pattern, Spider web sign,Bronchial wall thickening,Subpleural curvilinear line, Nodule,  Reticulation, Lymph node enlargement,Pleural effusion,Pericardial effusion |
| Zhou et al. [53] | China | 62 | RT-PCR | retrospective study | 52.8 | 39/23 | MDCT | 62 | GGO,Consolidation,GGO and reticular pattern,Vacuolar sign, Microvascular dilation sign, Fibrotic streaks, Subpleural line ,Subpleural transparent line ,Bronchial change,  Air bronchogram,Bronchus distortion,Pleural change,Thickening of pleura, Pleural retraction sign ,Pleural effusion |
| Xiong et al. [54] | China | 42 | RT-PCR | retrospective study | 49.5 | 25/17 | HRCT | 42 | Consolidation,Interstitial thickening,Air bronchograms,Fibrous stripes,Pleural effusion,  Lymph nodes changes |
| Cheng et al. [55] | China | 11 | RT-PCR and Chest CT | retrospective study | 50.3 | 8/3 | Without mention | 11 | GGO,Mixed GGO,Consolidation,Air bronchogram,Centrilobular nodules,Tree-in-bud sign,Reticular pattern,Subpleural linear opacity,Bronchial dilatation,Cystic change ,Pleural effusion |
| Guan et al . [56] | China | 53 | NAATs/  RT-PCR | retrospective study | 42 | 25/28 | Thin-section  Chest CT | 47 | GGO,Crazy-paving,Consolidation,Stripe,Air bronchogram,Pulmonary nodules,  Secondary tuberculosis,Cavity,Enlarged mediastinal lymph node,Pleural effusion |
| Han et al. [57] | China | 108 | RT-PCR | retrospective study | 45 | 38/70 | HRCT | 108 | Ground-glass opacity,Consolidation,Ground-glass opacity with consolidation,Vascular thickening,Crazy paving pattern Air bronchogram sign |
| Song et al. [58] | China | 51 | RT-PCR | retrospective study | 49 | 25/26 | Thin-section  Chest CT | 51 | GGO, consolidation, bilateral lung, peripheral, central, pleural effusion, pericardial  effusion, lymphadenopathy |
| Zhang et al. [59] | China | 140 (135 with chest  CT scan) | chest radiology and RT-PCR | retrospective study | 57 | 69/71 | Without mention | 134 | Bilateral lung |
| Guan et al. [60] | China | 1,099 (975 with  chest CT scan) | RT-PCR | retrospective study | 47 | 637/459 | Without mention | 840 | Bilateral lung, GGO,  interlobular septal  thickening |
| Bai et al. [61] | China | 256 | RT-PCR | retrospective study | NA | NA | Without mention | 219 | Ground-glass opacity,Consolidation,Linear opacity ,Interstitial change,Septal thickening,Fine reticular,opacity,Nodule,Vascular thickening,Bronchial wall thickening , Air bronchogram,Crazy-paving pattern,Halo sign ,Pleural thickening,Pleural effusion,Lymphadenopathy |
| Hu et al. [62] | China | 24 | RT-PCR | retrospective study | NA | NA | Without mention | 15 | ground-glass or patchy shadows stripe shadows in lungs, |

Abbreviations: GGO: Ground-glass opacities, CT: Computerized tomography, RT-PCR: Reverse transcription-polymerase chain reaction, HRCT: high-resolution computed tomography,

U-HRCT: Ultra-high-resolution CT, HD-DECT: High-dose dual-energy acquisition CT, LDCT: Low-dose CT.
