# Supplementary material for: Do COVID-19 CT features vary between patients from within and outside mainland China? Findings from a meta-analysis
Source: Front Public Health. 2022 Oct 14;10:939095. doi: 10.3389/fpubh.2022.939095 (PMC9616120; doi:10.3389/fpubh.2022.939095)
Supplement: Supplementary Table S2 — Risk of bias assessment of the included studies according to the Quality Assessment of Diagnostic Accuracy Studies (QUADAS) scale. [file Table_2.docx]

Supplement table 2: Risk of Bias assessment of the included studies according to the QUADAS-2

| Study | Risk of bias | | | | Applicability concerns | | |
| --- | --- | --- | --- | --- | --- | --- | --- |
|  | patient selection | Index  test | Reference  standard | Flow and  timing | patient selection | Index  test | Reference  standard |
| Caro-Dominguez et al. [32] | ☺ | ☺ | ☺ | ☺ | ☺ | ☺ | ☺ |
| Himoto et al. [33] | ☺ | ☺ | ☺ | ☺ | ☺ | ☺ | ☺ |
| Mohammdi et al. [34] | ☺ | ☺ | ☺ | ☺ | ☺ | ☺ | ☺ |
| Yoon et al. [35] | ☺ | ☺ | ☺ | ？ | ☺ | ☺ | ☺ |
| *Çinkooğlu* et al. [36] | ? | ☺ | ☺ | ？ | ☹ | ☺ | ☺ |
| Abrishami et al. [37] | ☺ | ☹ | ☺ | ☺ | ☺ | ☹ | ☺ |
| Achour et al. [38] | ☺ | ☺ | ☹ | ☺ | ☺ | ☺ | ☹ |
| Caruso et al. [39] | ☺ | ☺ | ☺ | ☺ | ☺ | ☺ | ☺ |
| Iwasawa et al. [40] | ☺ | ☺ | ☺ | ☺ | ☺ | ☺ | ☺ |
| Agostini et al. [41] | ☺ | ☺ | ☺ | ☺ | ☺ | ☺ | ☺ |
| Inui et al. [42] | ？ | ☺ | ？ | ☺ | ☹ | ☺ | ？ |
| Teich et al. [43] | ☺ | ☹ | ☺ | ☺ | ☺ | ☹ | ☺ |
| Korkmaz et al. [44] | ? | ☹ | ☺ | ☺ | ？ | ☹ | ☺ |
| Yoshimura et al. [45] | ☺ | ？ | ☺ | ☺ | ☺ | ？ | ☺ |
| Wu et al.[16] | ☺ | ? | ☺ | ☺ | ☺ | ☹ | ☺ |
| Ai et al.[17] | ☺ | ☺ | ☺ | ☺ | ☺ | ☺ | ☺ |
| Pan et al.[46] | ☺ | ☺ | ☹ | ☺ | ☺ | ☺ | ？ |
| Bernheim et al.[47] | ☺ | ☺ | ☺ | ☺ | ☺ | ☺ | ☺ |
| Shi et al.[48] | ☺ | ☺ | ☺ | ☹ | ☺ | ☺ | ☺ |
| Xu et al.[49] | ☺ | ☺ | ☺ | ☺ | ☺ | ☺ | ☺ |
| Xu et al.[50] | ☺ | ☹ | ☺ | ☺ | ☺ | ? | ☺ |
| Zhao et al.[51] | ☺ | ☺ | ☺ | ☺ | ☺ | ☺ | ☺ |
| Li et al.[52] | ☺ | ☺ | ☺ | ☺ | ☺ | ☺ | ☺ |
| Zhou et al.[53] | ☺ | ☺ | ☺ | ? | ☺ | ☺ | ☺ |
| Xiong et al.[54] | ☺ | ☺ | ☺ | ☹ | ☺ | ☺ | ☺ |
| Cheng et al.[55] | ☺ | ☺ | ☺ | ☺ | ☺ | ☺ | ☺ |
| Guan et al.[56]. | ☺ | ☺ | ? | ☺ | ☺ | ☺ | ☹ |
| Han et al.[57] | ☺ | ☺ | ☺ | ☺ | ☺ | ☺ | ☺ |
| Song et al.[58] | ☺ | ☺ | ☺ | ☺ | ☺ | ☺ | ☺ |
| Zhang et al.[59] | ☺ | ☺ | ☹ | ☺ | ☺ | ☺ | ? |
| Guan et al.[60] | ☺ | ☺ | ? | ☺ | ☺ | ☺ | ☹ |
| Bai et al.[61] | ☺ | ☺ | ? | ☺ | ☺ | ☺ | ☹ |
| Hu et al.[62] | ☺ | ☹ | ☺ | ☺ | ☺ | ? | ☺ |

☺Low Risk ☹High Risk ? Unclear Risk
